# Mortality prediction in patients with severe septic shock: a pilot study using a target metabolomics approach

**DOI:** 10.1038/srep20391

**Published:** 2016-02-05

**Authors:** Manuela Ferrario, Alice Cambiaghi, Laura Brunelli, Silvia Giordano, Pietro Caironi, Luca Guatteri, Ferdinando Raimondi, Luciano Gattinoni, Roberto Latini, Serge Masson, Giuseppe Ristagno, Roberta Pastorelli

**Affiliations:** 1 Politecnico di Milano, Milan, Italy; 2 IRCCS-Istituto di Ricerche Farmacologiche Mario Negri, Milan, Italy; 3 Dipartimento di Fisiopatologia Medico-Chirurgica e dei Trapianti, Fondazione IRCCS Ca’ Granda –Ospedale Maggiore Policlinico, Università degli Studi di Milano, Milan, Italy; 4 Dipartimento di Anestesia, Rianimazione, ed Emergenza Urgenza, Fondazione IRCCS Ca’ Granda – Ospedale Maggiore Policlinico, Università degli Studi di Milano, Milan, Italy; 5 Azienda Ospedaliera di Desio, Desio, Italy; 6 Azienda Ospedaliera Luigi Sacco, Milan, Italy

## Abstract

Septic shock remains a major problem in Intensive Care Unit, with high lethality and high-risk second lines treatments. In this preliminary retrospective investigation we examined plasma metabolome and clinical features in a subset of 20 patients with severe septic shock (SOFA score >8), enrolled in the multicenter Albumin Italian Outcome Sepsis study (ALBIOS, NCT00707122). Our purpose was to evaluate the changes of circulating metabolites in relation to mortality as a pilot study to be extended in a larger cohort. Patients were analyzed according to their 28-days and 90-days mortality. Metabolites were measured using a targeted mass spectrometry-based quantitative metabolomic approach that included acylcarnitines, aminoacids, biogenic amines, glycerophospholipids, sphingolipids, and sugars. Data-mining techniques were applied to evaluate the association of metabolites with mortality. Low unsaturated long-chain phosphatidylcholines and lysophosphatidylcholines species were associated with long-term survival (90-days) together with circulating kynurenine. Moreover, a decrease of these glycerophospholipids was associated to the event at 28-days and 90-days in combination with clinical variables such as cardiovascular SOFA score (28-day mortality model) or renal replacement therapy (90-day mortality model). Early changes in the plasma levels of both lipid species and kynurenine associated with mortality have potential implications for early intervention and discovering new target therapy.

Septic shock is the most severe complication of sepsis and is one of the main causes of death in intensive care units (ICUs)^1^. Although treatment has improved over the last decades, outcome is still poor and difficult to predict^2^. Indeed, the majority of patients with shock develops a multiple organ dysfunction syndrome (MODS), a condition in which organs not directly affected by the original infection become dysfunctional, and that ultimately represents the main cause of death^3^. The interrelationship between inadequate oxygen delivery to peripheral tissues, ischemia and reperfusion injury in the organs, hemodynamic instability, inflammation and development of MODS has been extensively investigated, but the molecular mechanisms which ultimately trigger tissue functional injury remain largely undetermined^2^.

In the recent, large, multicenter, randomized clinical trial ALBIOS (Albumin Italian Outcome Sepsis study, NCT00707122) that enrolled 1818 patients with severe sepsis or septic shock, 90-day mortality of the latter patients was 46.7%^4^. Given this high rate, similar to that observed in other comparable clinical studies^5^,^6^, a multimarker strategy may be helpful to better understand the complex pathogenesis of the disease and its evolution, especially for early risk stratification and implementation of personalized therapies. The use of emerging omics tools able of examining physiological responses at system level is particularly promising for complex and heterogeneous conditions such as septic shock.

Indeed, several recent studies have focused on investigating plasma metabolomic profiles as predictive signatures of ICU mortality in adult patients^7^,^8^,^9^,^10^. Different composite metabolite patterns have been identified with nuclear magnetic resonance (NMR) or mass spectrometry (MS). Although these methods have different intrinsic metabolomic coverage potential, they all clearly highlight the widespread metabolic abnormalities in patients with septic shock, and the interplay of several different biochemical pathways.

In the present study, we used a targeted mass spectrometry-based quantitative metabolomics approach focusing our attention on several series of metabolites, such as glycerophospholipids, aminoacids, biogenic amines and acylcarnitines, some of which have already been identified as part of key biochemical pathways in septic shock. Metabolic signatures showing alteration in circulating kynurenine, fatty acids, lysophosphatidylcholines species and /or carnitine esters have already been reported in different settings of septic shock patients^7^,^8^,^9^,^10^,^11^,^12^,^13^,^14^,^15^,^16^, pointing toward an overall derangement of energy circuits, lipid homeostasis and indicating that, probably, the magnitude of these changes might provide an indicator of disease severity.

We applied our metabolomic strategy on a selected subset of patients with severe septic shock (serum lactate concentration >4 mmol/L) with multiple organ failures (SOFA score >8) enrolled in the ALBIOS study. The severe condition under study is, fortunately, not frequent in septic shock patients, but it represents a pathological state associated with a mortality of 20% in the next 24 hours^17^.

Our explorative study was designed to provide absolute quantitative information on changes in plasma metabolite levels measured one day (initial acute phase) and one week after development of severe septic shock in ICU, and to relate these changes with mortality. The two time points were chosen to verify the hypothesis that the metabolic changes over the time period reflect not only initial clinical characteristics but also the progression of the disease and long-term survival. Association between metabolic patterns and mortality was assessed with univariate and multivariate analyses adjusted for clinical relevant variables.

The primary goal of this pilot investigation was to verify the feasibility of our metabolomic approach (e.g. metabolic profiles characterizing non-survivors) that is intended to be used in our ongoing larger scale study ShockOmics (NCT02141607), aimed at elucidating early multilevel markers signatures which could reveal the metabolic pathways involved, a necessary step for a actual target therapy.

## Results

### Clinical characteristics of the study population

**Figure 1:**
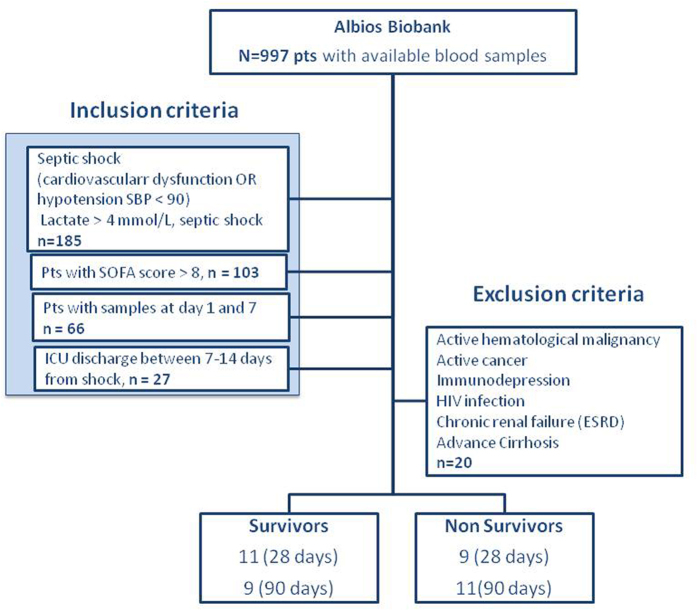
Flow chart, which displays the progress of patient selection from the ALBIOS biobank according to our inclusion/exclusion criteria.

All the patients with severe septic shock enrolled in the multicenter ALBIOS clinical trial^4^, and fulfilling the inclusion/exclusion criteria presented in details in the Methods section were analyzed. The data are summarized in the flow chart (Fig. 1). The baseline characteristics of these 20 patients are shown in Table 1. On day 28, mortality rate was 45% (9 patients), whereas on day 90 mortality rate was 55% (11 patients). Clinical variables on days 1 and 7 are shown in Table 2. In 11 patients (55%), source of infections was identified at site culture, including gram-negative (5 patients), gram-positive (2 patients) and both gram-negative and gram-positive bacterial infection (3 patients), as well as fungal infection (1 patient). In 9 of these patients (82%), antibiotic therapy empirically administered during the first 24 hours was appropriate. Patients were randomized to receive either 20% albumin and crystalloid solutions (13 patients) or crystalloid solutions alone (7 patients) for volume replacement. All the patient were treated according to the standard guidelines internationally accepted for the treatment of patients with severe sepsis or septic shock^18^.

### Time-course of plasma metabolites and association with mortality

We applied a mass spectrometry-based quantitative metabolomic profiling in order to unambiguously identify and quantify lipids, aminoacids, biogenic amines and acylcarnitines in the plasma of the study subjects. To ensure data quality and robust statistical analysis the following filtering criteria were applied: (i) metabolites measured with more than 20% missing data (no detectable peak) were excluded for any further data elaboration; (ii) metabolites for which the plasma concentration was below the limit of detection (<LOD) in at least ≥50% of analyzed samples were excluded.

In total, 137 metabolites were selected after quality control: 1 hexose, 21 amino acids, 14 biogenic amines, 3 acylcarnitines, 14 sphingomyelins (SM), 84 glycerophospholipids. Concentrations of all analyzed metabolites for each patient are expressed as μM and shown in Table S1 for day 1 (D1) and Table S2 for day 7 (D7).

**Figure 2:**
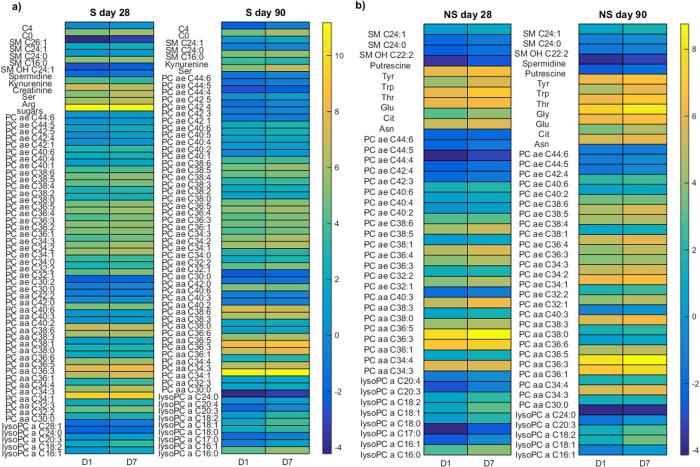
Heat maps of the metabolites (mean Log 2 μM) whose concentrations changed significantly from day 1 (D1) to day 7 (D7) in survivors (S) (**a**) and non-survivors (NS) (**b**) both considering mortality at 28 days and 90 days (Wilcoxon test p < 0.05, FDR < 0.15).

We first assessed by univariate analysis whether metabolite levels significantly changed from D1 to D7, after stratification by survival status. Figure 2 gives a pictorial overview of the changes of metabolite concentrations (row, mean Log2 μM) between D1 and D7 in survivors (S, Fig. 2a) and non-survivors (NS, Fig. 2b) (Wilcoxon test p < 0.05, FDR < 0.15, Table S3). Ten different species of lysophosphatidylcholines (LPC), 17 of diacyl-phosphatidylcholines (PC aa), 30 of acyl-alkyl phosphatidylcholines (PC ae), 2 of acylcarnitines (carnitine, C0; butyrylcarnitine, C4), 4 of long-chain sphingomyelins (SM) increased from D1 to D7 in 28- and 90-day survivors, while kynurenine (KYN) and two polyunsaturated diacyl-PC (PC aa C38:6, PC aa C40:6) decreased (Fig. 2a). NS at 28 or 90 days showed an overall increase from D1 to D7 for LPC, PC, and SM species. In NS, aminoacids doubled their plasma concentration together with putrescine and spermidine, the last one significant only in NS at day 90 (Fig. 2b).

Profiles of specific metabolites differed significantly between NS and S (Table 3). The majority of PC and LPC species showed lower values at D1 and D7 in NS when compared to S at day 28, whereas increased concentrations of acetylcarnitine (C2) and of KYN were observed in NS on D1 and D7, respectively. Significantly lower values of LPC and PC were measured at D1 and D7 in NS when compared to S at day 90. There were six lipid species comprising saturated long-chain LPC and polyunsaturated very long-chain PC, whose levels decreased at D7 in both NS at 28 or 90-days.

**Figure 3:**
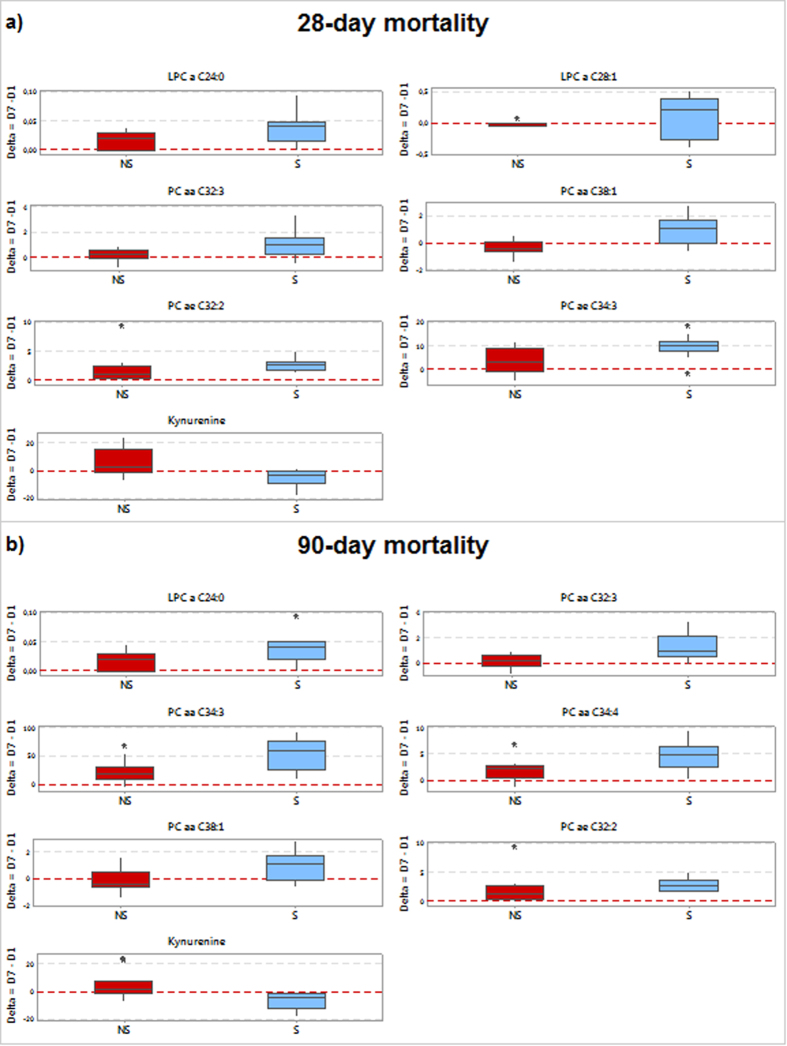
Comparison of the absolute differences in metabolite concentrations (μM) from day 1 to day 7 (Delta = D7–D1) in survivors (S) and non-survivors (NS) at 28 days (**a**) or 90 days (**b**). Distribution of differences is shown as box-plots, where the central mark is the median concentration, the edges of the box are the 25th and 75th percentiles, the outliers are defined as 1.5 times of interquartile range and highlighted by stars. Each plot represents a different metabolite. (Wilcoxon test p < 0.05, FDR < 0.15).

The changes of metabolites from D1 to D7, reported in Table S3, were then compared between S and NS. Significant differences in metabolite levels were found (Fig. 3) (Wilcoxon test p < 0.05, FDR < 0.15). A clear negative variation was observed for KYN, whereas LPC and PC (mainly low saturated long-chain species) showed a positive variation in 28- and 90-day survivors (Table S4).

### Association between metabolic patterns and mortality

**Figure 4:**
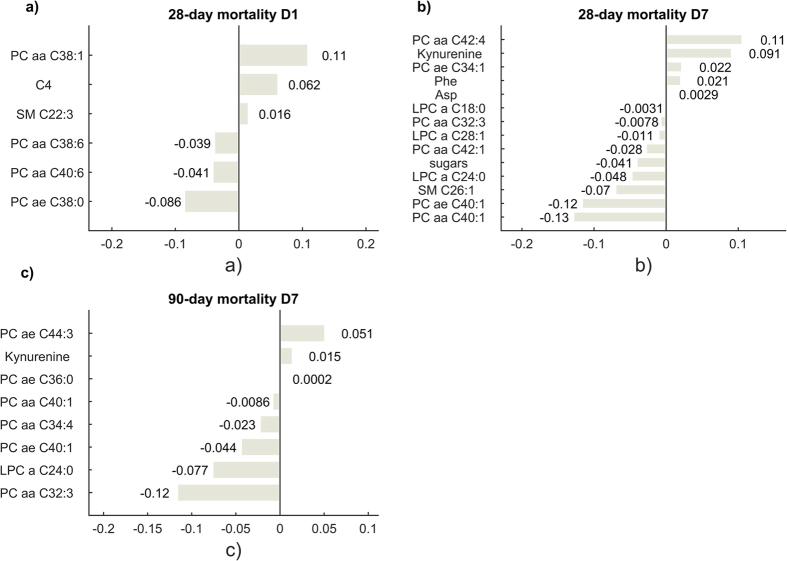
Elastic Net coefficients built on metabolites concentration at day 1 (**a**) and day 7 (**b**) for 28-day and 90 days mortality (**c**). The MSE computed for the models are 0.21 for (**a**), 0.26 for (**b**) and 0.27 for (**c**).

Multivariate analysis was performed using Elastic Net in order to predict the outcome of the patients at 28 or 90 days. Such technique permits to weigh the contribution added by each metabolite to the prediction model and to set to zero the coefficients of those that are not predictive (Fig. 4). The coefficients can be interpreted as in a linear regression model: the higher their absolute value, the higher their weight in the model. Similarly, a positive coefficient denotes a positive correlation with the event (i.e. death at day 28 or at day 90) and vice versa. On D1, PC aa C38:1 and C4 (butyryl-acylcarnitine) had a strong positive correlation with 28-day mortality, whereas PC aa C40:6 and PC ae C38:0 were inversely associated (Fig. 4a). On D7, KYN and PC aa C42:4 were positively correlated to 28-day mortality, while PC aa C40:1 and PC ae C40:1 are negatively correlated (Fig. 4b). Interestingly, long chain PC, LPC molecular species and KYN measured on D7 were consistently associated with 90-day mortality (i.e. KYN, PC ae C44:3, PC aa C32:3, PC ae C40:1, LPC a C24:0) (Fig. 4c).

### Integrated clinical and metabolomic determinants of mortality

We next checked for a possible redundancy of prognostic information among circulating metabolites and clinical variables.

**Figure 5:**
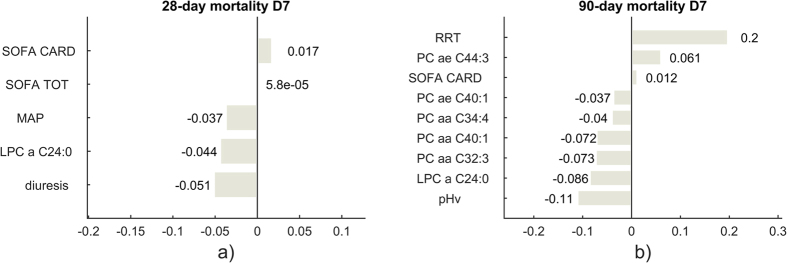
Elastic Net coefficients built on metabolites concentration and clinical parameters collected at day 7 for 28-day (**a**) and 90 days mortality (**b**). The MSE computed for the models are 0.23 for the 28-day one (**a**) and 0.24 for the 90-day (**b**). SOFA CARD: cardiovascular SOFA score; SOFA TOT: total SOFA score; MAP: mean arterial pressure; pHv: venous pH; diuresis (ml/die) collected at day 7; RRT: renal replacement therapy administered during the first 7 days of treatment.

Figure 5 shows the best elastic net regression models that considered both metabolites measured on D7 and clinical variables. For 28-day mortality (Fig. 5a), daily urinary output, plasma concentration of LPC a C24:0, and mean arterial pressure resulted negatively associated with the outcome, while the risk of death increased with the cardiovascular subcomponent of the SOFA score (need for vasoactive drugs). Interestingly, lower levels of LPC a C24:0 collected at D7 were the strongest predictors of 90-day mortality, whereas use of renal replacement therapy was positively associated with an increased risk of death (Fig. 5b). No reliable predictive models were obtained with metabolites and clinical parameters collected at D1.

## Discussion

This study is a preliminary investigation to characterize the metabolomic profiles of patients with severe septic shock and multiple organ failures at the time of its development, and to integrate them with the clinical manifestation characterizing this syndrome. It was meant as a preparatory study to the ongoing clinical trial (Shockomics, NCT02141607) aimed at elucidating early multilevel markers signatures that might help clinicians in understanding the particular pathophysiology characterizing a specific clinical phenotype of septic shock, in prioritizing individual patient treatment, and in developing therapy that acts on specific target of the syndrome.

The present study identifies several metabolomic alterations, previously reported in patients with severe sepsis and septic shock^7^,^8^,^9^,^10^,^11^,^12^,^15^,^16^, supporting the feasibility and the rationale underlying our pilot study design. Profiles of specific metabolites measured on day 1 and 7 differed markedly between survivors and non-survivors, and some metabolic features appeared to be associated with mortality. Though we cannot discuss the significance of every single metabolite, some general comments on the main metabolic pathways and their pathological relevance to septic shock are warranted.

Non-survivors were characterized by a significant elevation of the polyamine pool (spermidine, putrescine), from D1 to D7, especially in those who died at 90 days (Table S3 and Fig. 2). Since polyamines mediate the complex interplay between bacterial infection and the host immune response^19^,^20^ this might suggest an altered regulation of pathogen-host interactions in these patients. Moreover non-survivors had increased plasma level of glucogenic aminoacids (Table S3 and Fig. 2), in line with relative hepatic dysfunction occurring early in sepsis and consequent derangement in the hepatic gluconeogenesis as response to sepsis^21^,^22^.

A peculiarity of NS at days 28 and 90 was the significant increase from D1 to D7 of plasma KYN (Table S4 and Fig. 3). Indeed, KYN level at day 7 was almost doubled in 28-day NS compared to S. To date a clear relation has been made between accelerated tryptophan catabolism along the KYN pathway and inflammatory reactions. Bacterial products and proinflammatory cytokines upregulate indolamine 2,3-dioxygenase (IDO), the enzyme responsible for KYN production. IDO is critically involved in CD4^+^ and CD8^+^ effector T cell suppression as well as in generation and activation of regulatory T cells^23^,^24^. Recently, it has been shown that KYN plasma level might predict the development of sepsis in major trauma patients^25^, and its modulation has been associated to 28-day mortality in critically ill patients^8^. Increased production of KYN has been proposed to contribute to hypotension in sepsis^26^ and it has been associated with dysregulated immune response and impaired microvascular reactivity^27^. We can speculate that the lower KYN concentration found in survivors may represent a favorable host response trait. Indeed, our study support for the first time the hypothesis that higher KYN levels are associated with an increased long-term mortality during septic shock. However, whether kynurenine metabolism is a pathogenic factor in sepsis or rather an epiphenomenon needs further evaluation.

Decreased plasma level of PC and LPC species was a prominent component of the metabolic phenotype in NS (Fig. 3), in accordance with an overall lipidome alterations observed in sepsis and critically ill patients^7^,^13^,^14^,^15^,^16^. Already at 24 hours after the diagnosis of septic shock and ICU admission (D1), patients who did not survive within 90 days showed a marked decrease in PC species, containing long chain polyunsaturated fatty acid (LCPUFAs), such as PC C38:6, PC C38:4, PC C36:6, that persisted at D7 with further elongation/desaturation products. Since LCPUFAs reduce T-cell activation and dampen inflammation^28^, it might be speculated that a decrease in PC containing LCPUFAs can hamper their protective effects, including a concerted action of either withdrawing pro-inflammatory eicosanoids or incrementing anti-inflammatory eicosanoids. We can reasonably exclude dietary-derived influence on LCPUFAs, since the difference in their concentrations were present already at D1, and patients were all subjected to dietary support according to standard guidelines on the treatment of patients with severe sepsis or septic shock^18^.

A general explanation for these findings is that the lowered PC circulating level found in non-survivors might be due to reduced or unbalanced fatty acid substrates for their biosynthesis, consistent with a deregulated mitochondrial and/or peroxisomal beta-oxidation occurring early in sepsis^29^. Conversely, the decreased plasma acetylcarnitine observed in 28-day survivors compared to non-survivors would indicate a general more efficient use of substrates for energy production and for a mitochondrial damage probably reversible in survivors^30^.

A further bio-signature characterizing non-survivors was their reduction over time in circulating mono-saturated and saturated LPCs. Such changes in LPC concentration is in concordance with Park *et al.*^12^ who showed a similar downward trend of LPC in 28-day non-survivors as compared to survivors in sepsis. Decreased LPC have been also reported in septic patients compared to healthy controls^11^. The well-known pro-inflammatory activities of LPC^31^,^32^,^33^ sound in apparent contradiction with the poor outcome (and the lower LPC levels) observed in sepsis. The reduction in circulating LPC may simply reflect their enhanced conversion to lysophosphatidic acid, which is known to induce a multitude of cellular responses through its action on immunological relevant cells^34^. It is conceivable that LPC reduction may promote an excessive immune response with detrimental effect in those patients who will not survive^11^,^12^.

In the multiparameter model, low unsaturated long-chain PC species and LPC a C24:0 were associated with long-term mortality (90 days) together with circulating KYN. Moreover, LPC a C24:0 and PC aa C32:3 were negatively correlated to the event at 28 days and 90 days in combination with clinical variables, in particular, the cardiovascular SOFA score for the 28-day mortality model and the necessity of renal replacement therapy (e.g. continuous veno-venous hemofiltration, CVVH, or dialysis) during the 7 day ICU staying for the 90-day mortality model. The recurrent decrease in LPC a C24:0 may denote an alteration in very long chain fatty acids, such as lignoceric acid as preferred substrates for the peroxisomal beta-oxidation. Consequently, a down-regulation of the peroxisomal lignoceryl-CoA ligase activity might be hypothesized. Indeed, reduced peroxisome proliferator-activated receptor α (PPARα) expression has been associated with decreased survival in pediatric patients with septic shock^35^. Our findings suggest a multifactorial origin for such abnormal phospholipids metabolism, in which dysregulation of phospholipases, catabolism of LPC, peroxisomal dysfunction, imbalance in the levels of saturated/unsaturated fatty acids could all be involved. However, the underlying molecular mechanisms potentially regulating the circulating PC and LPC species in survivors as compared to non-survivors remain unclear and warrant further investigations.

Our study has several limitations. First, a targeted approach restricts, by its nature, the panel of candidate markers and focuses only on few metabolic pathways. Second, the sample size is limited, and confirmatory studies are necessary. Since many patients with such severity of septic shock do not reach day 7 of ICU staying, we end up with a limited number of patients fulfilling our study criteria. To note that the primary purpose of this investigation was to examine the feasibility of our metabolomics approach in determining changes in circulating metabolites able to characterize the progress of septic shock condition and to reveal the involved pathways.

Third, we measured metabolites at two time points only within one week from the diagnosis of septic shock, and metabolites with temporal changes out of this time window might provide a more precise insight for the clinical progression of the disease. Nevertheless, we identified a combination of circulating metabolites altered during the early course of severe septic shock and associated with mortality.

This preliminary investigation was therefore very informative in capturing possible evolution and variations of metabolic signatures during a full blown, durable and well-established pathophysiologic manifestation of severe septic shock. Focusing on a homogeneous group of patients rather than on a larger number of scattered phenotypes allowed for a better control of potentially confounding factors. Therefore, the metabolic changes observed in our samples pertains more closely to the selected pathophysiological condition and it should be proved in a larger cohort by including different phenotypes such as not only the severe patients and multimarkers information such as multi-omics data.

## Conclusions

In conclusion, the data presented here confirm the feasibility of our approach in determining changes circulating metabolites able to characterize the progress of septic shock condition. Our results are in line with recent findings indicating that lipid homeostasis and tryptophan catabolism might influence mortality in septic shock. The association of early changes in the plasma levels of both lipid species and KYN with mortality, with possible implications for early intervention is the most important result of our study Although our analyses cannot determine causality, it suggests that alterations in KYN and lipid species might represent not only risk factors for patients with severe septic shock but important pathophysiologic mechanisms deserving further investigations.

## Materials and Methods

### Study design, patients and clinical data

This pilot retrospective metabolomic investigation was a substudy of the multicenter, randomized Albumin Italian Outcome Sepsis (ALBIOS) clinical trial, which enrolled patients with severe septic or septic shock from 100 ICU in Italy (NCT00707122). Details of the ALBIOS study and protocol are fully described in the original article^4^.

Inclusion criteria for the present study were the presence of septic shock^36^ (as defined by the presence of a proved or suspected infection in at least one site; two or more of the signs of systemic inflammatory reaction syndrome; the presence of an acute sepsis-related cardiovascular dysfunction or the presence of a systolic blood pressure < 90 mmHg), serum concentrations of lactate >4 mmol/L, a total SOFA score >8, , and with plasma samples available at day 1 (D1) and day 7 (D7) after diagnosis of septic shock in the ALBIOS biobank. In addition, we consider only patients remaining in ICU until 7 to 14 days from shock (until either ICU discharge or death). This allows elucidating with good accuracy and solidity the possible association of metabolite profiles with patient outcome, as close to blood sample collection (day 7). Exclusion criteria included the presence of active hematological malignancy or cancer, immunodepression, HIV infection, chronic renal failure, or advanced cirrhosis. Such inclusion and exclusion criteria were in accordance with those of the ongoing multicenter clinical study, ShockOmics (NCT02141607), and the current study represents a preliminary investigation.

Only 20 among the 997 patients enrolled in ALBIOS trial and with plasma samples stored in the biobank fulfilled the inclusion criteria (see Fig. 1). The following demographic, clinical and laboratory variables were considered: (i) information about the patient collected at ICU admission: age (years), sex, body mass index, pathological conditions before hospitalization (presence of hepatic insufficiency, renal insufficiency, respiratory pathologies, immunocompromised state, cardiovascular pathologies), Simplified Acute Physiology Score (SAPS), duration of primary infection; (ii) hemodynamic parameters: heart rate (bpm), mean arterial pressure (mmHg), venous central pressure (mmHg), daily urinary output (ml/die); (iii) ventilation parameters: the need for ventilatory support, positive end-expiratory pressure (cmH_2_0), FiO_2_ (%); (iv) blood gas analysis: central venous O_2_ saturation, venous partial pressure of CO_2_, arterial partial pressure of CO_2_, arterial partial pressure of O_2_, central venous partial pressure of O_2_, arterial and venous pH; (v) laboratory and clinical parameters: serum concentrations of creatinine (mg/dL), biliuribin (mg/dL), and lactate (mmol/L), platelet count (x10^3^ cells/mm^3^); (vi) Sequential Organ Failure Assessment Score (SOFA)^37^, total SOFA score and the sub-scores relating to the respiratory, coagulation, hepatic, cardiovascular, and renal systems; (vii) outcome: mortality at 28 and 90. Details of study population are reported in Table 1 and Table 2.

Outcome was defined as mortality at 28 and 90 days. For each patient, plasma samples were available at day 1 (acute state, D1) and at day 7 (steady state; D7) after diagnosis of septic shock for the present metabolomics analysis.

### Target plasma metabolomics analysis

A targeted quantitative approach using a combined direct flow injection and liquid chromatography (LC) tandem mass spectrometry (MS/MS) assay (AbsoluteIDQ 180 kit, Biocrates, Innsbruck, Austria) was applied for the metabolomics analysis to EDTA-plasma samples stored at −80 °C at the ALBIOS-biobank. The method combines derivatization and extraction of analytes with the selective mass-spectrometric detection using multiple reaction monitoring (MRM) pairs. Isotope-labeled internal standards are integrated into the platform for metabolite absolute quantification. This strategy allows simultaneous quantification of 186 metabolites (40 amino acids and biogenic amines, 40 acylcarnitines, 90 glycerophospholipids, 15 sphingomyelins, 1 monosaccharide). A summary of the metabolomic analysis and the list of all the measurable metabolites are provided in Supplemental Methods.

### Statistical analysis

In the present analysis, patients were analyzed according to their survival status at 28 and 90 days after study enrollment. All quantified metabolites were considered in the analysis, together with the clinical parameters available at study enrollment and those related to patient condition at day 1 and at day 7 (Tables 1 and 2).

The comparisons between survivors (S) and non-survivors (NS) were performed by unpaired Wilcoxon Test for clinical parameters and metabolite concentrations (μM). The changes of the parameters from day 1 to day 7 were evaluated by means of the paired Wilcoxon signed rank test separately for the S and NS groups. Finally, the variations of the metabolites concentrations were compared between S and NS group by unpaired Wilcoxon test. We calculated also the false discovery rate (FDR) to overcome the problem of the large number of statistical comparisons. To compute the FDR, we used a bootstrapping technique of oversampling with replacement to obtain a sample size of 20 patients for each group (S and NS).

Multivariate analysis was performed using the Elastic Net technique, a shrinkage regression method effective in case of several highly correlated variables^38^. The Elastic Net performs continuous variable selection causing some of the regression coefficients to be exactly zero, thus reducing the variance of the regression estimates by eliminating redundant predictors. Furthermore, the subset of variables corresponding to non-zero coefficients can be considered as the predictors mainly associated to the outcome. Firstly, metabolomics data and clinical parameters were normalized to have unitary variance and zero mean. Different models were built with 2, 4, 5 and 10-fold cross validation (CV) for every data set analyzed, and the model with the minimum Mean Squared Error (MSE) was selected. As for the FDR, we used a bootstrapping technique of oversampling with replacement to obtain a sample size of 20 patients for each group (S and NS) by maintaining the same percentage of events. The outcome (survivors = 0, non-survivors = 1) was considered as output of the model. The best model was selected among the different CV models based on one-standard error rule.

## Additional Information

**How to cite this article**: Ferrario, M. *et al.* Mortality prediction in patients with severe septic shock: a pilot study using a target metabolomics approach. *Sci. Rep.* **6**, 20391; doi: 10.1038/srep20391 (2016).

## Supplementary Material

Supplementary Information

## Figures and Tables

**Table 1:** Characteristics at study enrollment and in the two groups of patients (S: survivors; NS: non survivors at 90 days).

**Clinical variable**	**ALL**	**S**	**NS**	**pValue**
# patients	20	9 (45%)	11 (55%)	
Age (yrs)	66.1 ± 13.9	61.3 ± 15.2	69.9 ± 12	n.s.
BMI	27 ± 4	27.6 ± 4	26.5 ± 4	n.s.
Sex (males)	65%	44.4%	81.8%	n.s.
Coexisting diseases:				
Liver disease (#pts)	0	0	0	n.s.
COPD (#pts)	2	1	1	n.s.
Chronic renal failure (#pts)	0	0	0	n.s.
Immunodeficiency (#pts)	0	0	0	n.s.
Congestive or ischemic heart disease (#pts)	2	1	1	n.s.
SAPS II score	57.5 ± 14.9	54.8 ± 17.9	61.1 ± 9.4	n.s.
SOFA score	11.4 ± 1.9	10.5 ± 1.5	12.1 ± 2	n.s.
MAP (mmHg)	68.6 ± 14.4	70.7 ± 16.7	66.8 ± 12.7	n.s.
CVP (mmHg)	12.6 ± 6	13.3 ± 6	12.1 ± 6	n.s.
PEEP (cmH_2_O)	7.4 ± 3.7	6.2 ± 4.3	8.3 ± 3	n.s.
FiO_2_ (%)	65 ± 19.3	63.3 ± 17.4	66.4 ± 21.6	n.s.
ScvO_2_ (%)	73.2 ± 9.6	70.3 ± 10.4	75.6 ± 8.1	n.s.
PvCO_2_ (mmHg)	43.3 ± 6.9	45.6 ± 6.3	41.3 ± 6.8	n.s.
PaCO_2_ (mmHg)	38.1 ± 6.9	38 ± 5	38.2 ± 8.9	n.s.
Serum lactate (mmol/L)	7.3 ± 2.4	7.9 ± 2.7	6.8 ± 2.2	n.s.
Platelet count (x10^3^ cells/mm^3^)	79.4 ± 51.6	79.8 ± 47.4	79.2 ± 57.4	n.s.
Serum Creatinine (mg/dL)	2.3 ± 0.9	2.3 ± 0.9	2.2 ± 1	n.s.
Serum Biliuribin (mg/dL)	2.6 ± 2.9	1.1 ± 0.5	3.8 ± 3.6	0.018
Arterial pH	7.33 ± 0.91	7.32 ± 0.1	7.34 ± 0.1	n.s.
PaO_2_ (mmHg)	128.6 ± 61.2	135.8 ± 61.6	122.7 ± 63.2	n.s.
Venous pH	7.30 ± 0.09	7.28 ± 0.11	7.32 ± 0.07	n.s.
PvO_2_ (mmHg)	43.2 ± 8.6	42.4 ± 10.6	43.6 ± 6.6	n.s.

Data are presented as mean ± SD or frequency. n.s.=not significant. BMI, body mass index; COPD, Chronic obstructive pulmonary disease; MAP, mean arterial pressure; CVP, central venous pressure; PEEP, positive end-expiratory pressure; FiO_2_, inspired oxygen fraction; ScvO2, central venous O_2_ saturation; PvCO_2_, venous partial pressure of CO_2_; PaCO_2_, arterial partial pressure of CO_2_; PaO_2_, arterial partial pressure of O_2_; PvO_2_, central venous partial pressure of O_2_.

**Table 2:** Clinical and laboratory variables at day 1 and day 7 for the 20 patients.

**Clinical variable**	**D1**	**D7**
Heart Rate (bpm)	103.5 ± 20.3	85.4 ± 10.3
MAP (mmHg)	75.7 ± 14.3	87.3 ± 15.8
CVP (mmHg)	11.7 ± 5.1	7.9 ± 3.7
Presence of ventilatory support (#pts)	20	13
PEEP (cmH_2_O)	8.8 ± 2.6	6.6 ± 4.6
FiO_2_ (%)	57.8 ± 15.2	44 ± 16.1
ScvO_2_ (%)	76.3 ± 9.3	76 ± 6.8
PvCO_2_ (mmHg)	47.3 ± 5.9	48.9 ± 6.2
PaCO_2_ (mmHg)	43.4 ± 6.7	43.3 ± 6.1
Serum lactate (mmol/L)	4 ± 2.2	1.8 ± 1.4
SOFA score	11.6 ± 2.7	6.8 ± 4
Respiratory SOFA score	2.4 ± 1	1.6 ± 0.9
Coagulation SOFA score	2.4 ± 1.1	1.6 ± 1.1
Hepatic SOFA score	1.3 ± 1.1	1.3 ± 1.4
Cardiovascular SOFA score	3.4 ± 0.9	0.6 ± 1.2
Renal SOFA score	2.1 ± 1.2	1.6 ± 1.6
Diuresis (ml/die)	2006.2 ± 1323.7	2944.7 ± 1870.2
Platelet count (×10^3^ cells/mm^3^)	63.8 ± 50.8	96.9 ± 55.9
Creatinine (mg/dL)	2.3 ± 1.1	1.8 ± 1.5
Biliuribin (mg/dL)	3 ± 3.5	4.6 ± 7.6
Arterial pH	7.4 ± 0.1	7.4 ± 0.1
PaO_2_ (mmHg)	115.3 ± 48.3	115 ± 44.9
Venous pH	7.4 ± 0.1	7.4 ± 0.1
PvO_2_ (mmHg)	45.1 ± 5.9	42.9 ± 6.3
Use of renal replacement therapy (#pts)	3	3

Data are presented as mean ± SD. No significant differences were found between D1 and D7. MAP, mean arterial pressure; CVP, central venous pressure; PEEP, positive end-expiratory pressure; FiO_2_, inspired oxygen fraction; ScvO_2_, central venous O_2_ saturation; PvCO_2_, venous partial pressure of CO_2_; PaCO_2_, arterial partial pressure of CO_2_; PaO_2_, arterial partial pressure of O_2_; PvO_2_, central venous partial pressure of O_2_.

**Table 3:** Metabolite level comparison between survivors (S) and non-survivors (NS) at day 1 and at day 7 for the 28-day and 90-day mortality.

	**METABOLITE**	**S**	**NS**	**pValue**	**FDR**	**NS vs S**	
28 day mortality	D1	LPC a C16:1	0.657 (0.334, 0.970)	0.313 (0.291, 0.591)	0.040	0.003	↓
		PC aa C30:2	0.005 (0.005, 0.026)	0.099 (0.017, 0.147)	0.046	0.005	↑
		PC aa C38:1	0.757 (0.499, 0.936)	1.025 (0.886, 1.645)	0.028	<10^−6^	↑
		PC aa C38:6	164.022 (131.032,174.123)	92.206 (47.999,137.945)	0.033	<10^−6^	↓
		PC ae C38:0	2.633 (2.418, 3.202)	1.712 (1.124, 2.241)	0.015	<10^−6^	↓
		SM C20:2	0.095 (0.068, 0.121)	0.055 (0.043, 0.088)	0.048	0.001	↓
		C2	5.080 (3.369, 8.774)	11.066 (8.189, 21.852)	0.048	0.028	↑
	D7	LPC a C16:0*	47.046 (24.384, 58.821)	18.150 (14.455, 33.212)	0.048	0.001	↓
		LPC a C18:0*	10.807 (6.188, 14.137)	5.684 (3.502, 7.111)	0.040	0.003	↓
		LPC a C24:0*	0.096 (0.086, 0.108)	0.066 (0.062, 0.085)	0.010	<10^−6^	↓
		PC aa C32:3*	3.486 (2.769, 4.240)	2.019 (1.807, 2.486)	0.028	0.001	↓
		PC aa C34:4*	8.604 (6.879, 11.438)	4.150 (3.464, 5.720)	0.028	<10^−6^	↓
		PC aa C36:4*	615.675(487.555,717.315)	369.822(340.428,463.466)	0.048	<10^−6^	↓
		PC ae C34:3	26.979 (20.127, 31.112)	18.013 (15.057, 21.835)	0.048	0.002	↓
		PC ae C40:1*	1.633 (1.209, 1.706)	0.844 (0.770, 1.230)	0.010	0.012	↓
		PC ae C42:4	0.788 (0.679, 0.892)	0.608 (0.487, 0.663)	0.040	<10^−6^	↓
		Kynurenine	7.680 (4.965, 8.735)	12.000 (8.745, 23.800)	0.012	<10^−6^	↑
90 day mortality	D1	PC aa C36:6	2.109 (1.630 , 2.435)	1.357 (0.957, 1.712)	0.033	0.0001	↓
		PC aa C38:4	225.147 (180.141 , 297.671)	142.022 (110.846, 217.886)	0.040	0.0106	↓
		PC aa C38:6	165.706 (137.786, 182.901)	107.441 (57.345, 152.421)	0.033	0.0002	↓
	D7	LPC a C16:0*	20.493 (6.241, 28.505)	12.308 (5.056, 16.828)	0.048	0.0002	↓
		LPC a C16:1	0.657 (0.314, 0.967)	0.467 (0.304, 0.610)	0.040	<10^−6^	↓
		LPC a C18:0*	4.363 (1.679, 6.895)	2.707 (1.658, 4.038)	0.040	0.0002	↓
		LPC a C24:0 *	0.054 (0.051, 0.073)	0.057 (0.045, 0.066)	0.008	<10^−6^	↑
		PC aa C32:2	22.073 (15.415, 30.845)	18.334 (12.289, 21.833)	0.033	0.0007	↓
		PC aa C32:3*	2.629 (1.924, 3.040)	1.826 (1.480, 2.195)	0.008	<10^−6^	↓
		PC aa C34:3	74.941 (47.836, 87.092)	51.173 (38.172, 65.232)	0.040	0.0002	↓
		PC aa C34:4*	5.129 (2.8154, 5.778)	2.938 (2.173, 3.918)	0.006	0.0001	↓
		PC aa C36:4*	827.639 (414.531, 919.064)	461.559 (352.353, 563.834)	0.023	0.0008	↓
		PC aa C36:5	51.571 (28.557, 62.269)	24.106 (19.734, 33.696)	0.019	0.0004	↓
		PC aa C36:6	2.110 (1.630, 2.435)	1.357 (0.957, 1.712)	0.023	0.0001	↓
		PC ae C32:2	4.658 (3.829, 5.043)	3.726 (3.195, 4.541)	0.040	0.0003	↓
		PC ae C38:0	2.633 (2.293, 3.450)	2.167 (1.221, 2.341)	0.033	0.0041	↓
		PC ae C40:1*	0.911 (0.654, 1.276)	0.683 (0.426, 1.031)	0.008	<10^−6^	↓
		PC ae C42:5	1.353 (1.068, 1.924)	1.138 (0.958, 1.920)	0.048	0.0006	↓
		PC ae C44:6	0.462 (0.296, 0.612)	0.401 (0.275, 0.552)	0.048	0.0004	↓

Only significant results are reported (p < 0.05, FDR < 0.15). Plasma concentrations are expressed in μM and shown as median (25, 75 percentiles). The arrows indicate that the metabolite concentration in NS group is lower (↓) or higher (↑) with respect to S group. The symbol * marks significant metabolites in both mortality groups.
