# Supplementary material for: Mortality prediction in patients with severe septic shock: a pilot study using a target metabolomics approach
Source: Sci Rep. 2016 Feb 5;6:20391. doi: 10.1038/srep20391 (PMC4742912; doi:10.1038/srep20391)
Supplement: Supplementary Information [file srep20391-s1.pdf]

Supplementary Information

Mortality prediction in patients with severe septic shock: a pilot study using a target metabolomics approach.

Manuela Ferrario[1#*], Alice Cambiaghi [1#], Laura Brunelli [2#], Silvia Giordano[2], Pietro Caironi [3,4], Luca Guatteri[5], Ferdinando Raimondi[6], Luciano Gattinoni [3,4], Roberto Latini [2], Serge Masson [2], Giuseppe Ristagno [2] and Roberta Pastorelli [2*]

[1] Politecnico di Milano, Milan, Italy

[2] IRCCS-Istituto di Ricerche Farmacologiche Mario Negri, Milan, Italy

[3] Dipartimento di Fisiopatologia Medico-Chirurgica e dei Trapianti, Fondazione IRCCS Ca' Granda –Ospedale Maggiore Policlinico, Università degli Studi di Milano, Milan, Italy

[4] Dipartimento di Anestesia, Rianimazione, ed Emergenza Urgenza, Fondazione IRCCS Ca' Granda – Ospedale Maggiore Policlinico, Università degli Studi di Milano, Milan, Italy

[5] Azienda Ospedaliera di Desio, Desio, Italy

[6] Azienda Ospedaliera Luigi Sacco, Milan, Italy

Supplemental material list:

Supplemental Methods

Supplemental Table S1

Supplemental Table S2

Supplemental Table S3

Supplemental Table S4

Supplemental Methods

Target Metabolomics

Targeted metabolomics analysis of plasma samples was performed using the Biocrates AbsoluteIDQ^{TM} p180 kit (Biocrates Life Science AG, Innskruck, Austria). This validated targeted assay allows for simultaneous detection and quantification of metabolites in plasma samples (10 microL) in a high-throughput manner. The plasma samples were processed following the instructions by the manufacturer and analyzed on a triple-quadropole mass spectrometer (AB SCIEX triple-quad 5500) operating in the multiple reaction monitoring (MRM-MS) mode. The assay is based on PITC (phenylisothiocyanate)-derivatization in the presence of internal standards for the analysis of aminoacids and biogenic amines resolved and quantified by liquid chromatography- tandem mass spectrometry (LC-MS/MS) using scheduled MRMs. Subsequent flow injection analysis tandem mass spectrometry (FIA-MS/MS) is performed to analyze acylcarnitines, glycerophospholipids, hexose. MRM detection is used for quantification applying spectra parsing algorithm integrated into the MetIQ software (Biocrates Life Science AG, Innskruck, Austria). Concentrations are calculated and evaluated by comparing measured analytes in a defined extracted ion count section to those of specific labeled internal standards or non-labeled ones, provided by the kit. The measurements are made in a 96-well format. Seven calibration standards, four quality control samples, three zero samples (PBS) and one blank (solvents) are integrated into the plate.

For glycerophospholipids, the precise position of the double bonds and the distribution of the carbon atoms in different fatty acid side chains cannot be determined with this technology. Lipid side-chain composition is abbreviated as C*x*:*y*, where *x* denotes the number of carbons in the side chain and *y* the number of double bonds. The nature of fatty acids linkage is expressed as aa for diacyl or ae for acyl-alkyl. For example, PCaaC32:1 denotes diacyl-phosphatidylcholine with 32 carbons in the two fatty acids side chains and a single double bond in one of them. Triplicate

analysis of plasma samples yielded average coefficient of variance (CV) below 0.09 for the metabolites measured, being the lowest for aminoacids (CV% 0.06) the highest for sphingomyelins (CV% 1.33). The list of all the measurable metabolites is provided below.

Metabolites measured by MRM-MS

Acylcarnitines

C0	Carnitine	C10:1	Decenoylcarnitine
C2	Acetylcarnitine	C10:2	Decadienylcarnitine
C3	Propionylcarnitine	C12	Dodecanoylcarnitine
C3:1	Propenoylcarnitine	C12:1	Dodecenoylcarnitine
C3-OH	Hydroxypropionylcarnitine	C12-DC	Dodecanedioylcarnitine
C4	Butyrylcarnitine	C14	Tetradecanoylcarnitine
C4:1	Butenylcarnitine	C14:1	Tetradecenoylcarnitine
C4-OH (C3-DC)	Hydroxybutyrylcarnitine	C14:1-OH	Hydroxytetradecenoylcarnitine
C5	Valerylcarnitine	C14:2	Tetradecadienylcarnitine
C5:1	Tiglylcarnitine	C14:2-OH	Hydroxytetradecadienylcarnitine
C5:1-DC	Glutaconylcarnitine	C16	Hexadecanoylcarnitine
C5-DC (C6-OH)	Glutarylcarnitine* (Hydroxyhexanoylcarnitine)	C16:1	Hexadecenoylcarnitine
C5-M-DC	Methylglutarylcarnitine	C16:1-OH	Hydroxyhexadecenoylcarnitine
C5-OH (C3-DC-M)	Hydroxyvalerylcarnitine (Methylmalonylcarnitine)	C16:2	Hexadecadienylcarnitine
C6 (C4:1-DC)	Hexanoylcarnitine (Fumarylcarnitine)	C16:2-OH	Hydroxyhexadecadienylcarnitine
C6:1	Hexenoylcarnitine	C16-OH	Hydroxyhexadecanoylcarnitine
C7-DC	Pimelylcarnitine	C18	Octadecanoylcarnitine
C8	Octanoylcarnitine	C18:1	Octadecenoylcarnitine
C9	Nonaylcarnitine	C18:1-OH	Hydroxyoctadecenoylcarnitine
C10	Decanoylcarnitine	C18:2	Octadecadienylcarnitine

Amino Acids and Biogenic Amines

Ala	Alanine	Ac-Orn	Acetylornithine
Arg	Arginine	ADMA	Asymmetric dimethylarginine
Asn	Asparagine	SDMA	Symmetric dimethylarginine
Asp	Aspartate	total DMA	Total dimethylarginine
Cit	Citrulline	alpha-AAA	alpha-Aminoadipic acid
Gln	Glutamine	Carnosine	Carnosine
Glu	Glutamate	Creatinine	Creatinine
Gly	Glycine	Histamine	Histamine
His	Histidine	Kynurenine	Kynurenine
Ile	Isoleucine	Met-SO	Methioninesulfoxide
Leu	Leucine	Nitro-Tyr	Nitrotyrosine
Lys	Lysine	OH-Pro	Hydroxyproline

Sphingolipids

SM (OH) C14:1	SM C18:0	SM (OH) C22:1	SM (OH) C24:1
SM C16:0	SM C18:1	SM (OH) C22:2	SM C26:0
SM C16:1	SM C20:2	SM C24:0	SM C26:1
SM (OH) C16:1	SM C22:3	SM C24:1	

Glycerophospholipids

lysoPC a C14:0	PC aa C34:1	PC aa C42:0	PC ae C38:2
lysoPC a C16:0	PC aa C34:2	PC aa C42:1	PC ae C38:3
lysoPC a C16:1	PC aa C34:3	PC aa C42:2	PC ae C38:4
lysoPC a C17:0	PC aa C34:4	PC aa C42:4	PC ae C38:5
lysoPC a C18:0	PC aa C36:0	PC aa C42:5	PC ae C38:6
lysoPC a C18:1	PC aa C36:1	PC aa C42:6	PC ae C40:1
lysoPC a C18:2	PC aa C36:2	PC ae C30:0	PC ae C40:2
lysoPC a C20:3	PC aa C36:3	PC ae C30:1	PC ae C40:3
lysoPC a C20:4	PC aa C36:4	PC ae C30:2	PC aa C40:4
lysoPC a C24:0	PC aa C36:5	PC ae C32:1	PC ae C40:5
lysoPC a C26:0	PC aa C36:6	PC ae C32:2	PC ae C40:6
lysoPC a C26:1	PC aa C38:0	PC ae C34:0	PC ae C42:0
lysoPC a C28:0	PC aa C38:1	PC ae C34:1	PC ae C42:1
lysoPC a C28:1	PC aa C38:3	PC ae C34:2	PC ae C42:2
PC aa C24:0	PC aa C38:4	PC ae C34:3	PC ae C42:3
PC aa C26:0	PC aa C38:5	PC ae C36:0	PC ae C42:4
PC aa C28:1	PC aa C38:6	PC ae C36:1	PC ae C42:5
PC aa C30:0	PC aa C40:1	PC ae C36:2	PC ae C44:3
PC aa C30:2	PC aa C40:2	PC ae C36:3	PC ae C44:4
PC aa C32:0	PC aa C40:3	PC ae C36:4	PC ae C44:5
PC aa C32:1	PC aa C40:4	PC ae C36:5	PC ae C44:6
PC aa C32:2	PC aa C40:5	PC ae C38:0	
PC aa C32:3	PC aa C40:6	PC ae C38:1	

Hexoses

H1	Hexose

Table S1 – Plasma concentration (µM) of quantified metabolites at day 1 for each patient. Survival status at 28 days and 90 days is also reported (S, survivor; NS, non survivor).

Metabolite D1	#1	#2	#3	#4	#5	#6	#7	#8	#9	#10	#11	#12	#13	#14	#15	#16	#17	#18	#19	#20
**28 day survival**	**S**	**S**	**NS**	**NS**	**NS**	**NS**	**S**	**S**	**S**	**S**	**NS**	**NS**	**S**	**S**	**S**	**NS**	**NS**	**S**	**NS**	**S**
**90 day survival**	**NS**	**S**	**NS**	**NS**	**NS**	**NS**	**S**	**NS**	**S**	**S**	**NS**	**NS**	**S**	**S**	**S**	**NS**	**NS**	**S**	**NS**	**S**
**LPC a C16:0**	12.308	7.528	13.655	4.899	28.277	5.527	5.857	126.81	29.717	6.368	10.370	4.339	28.175	5.650	20.493	13.126	17.885	29.495	4.883	26.565
**LPC a C16:1**	0.467	0.310	0.587	0.304	0.604	0.250	0.308	3.733	0.900	0.315	0.305	0.313	1.213	0.390	0.972	0.643	0.514	0.966	0.244	0.657
**LPC a C17:0**	0.233	0.160	0.245	0.134	0.371	0.127	0.108	1.497	0.418	0.121	0.129	0.129	0.389	0.105	0.466	0.398	0.259	0.434	0.124	0.413
**LPC a C18:0**	2.965	1.698	3.869	1.810	4.715	1.546	1.933	27.255	11.051	1.620	1.944	1.607	6.423	1.501	6.753	2.707	4.094	7.320	1.311	4.364
**LPC a C18:1**	2.303	1.917	4.377	2.778	3.995	1.936	1.707	32.610	6.319	2.273	2.470	2.570	4.382	1.504	3.997	7.747	3.395	4.625	1.827	3.481
**LPC a C18:2**	1.591	1.716	3.163	3.908	3.172	1.509	1.432	18.028	3.972	1.454	1.807	2.068	3.218	1.438	2.005	9.460	2.774	2.922	1.163	2.186
**LPC a C20:3**	0.411	0.302	0.520	0.331	0.299	0.291	0.289	2.082	0.558	0.152	0.243	0.276	0.471	0.174	0.292	0.619	0.392	0.257	0.258	0.305
**LPC a C20:4**	0.663	0.700	1.741	0.799	1.194	0.577	0.535	5.442	1.584	0.596	0.473	0.657	2.074	0.544	1.104	1.881	1.038	1.252	0.316	2.752
**LPC a C24:0**	0.048	0.053	0.082	0.057	0.045	0.066	0.046	0.061	0.053	0.054	0.037	0.047	0.071	0.048	0.061	0.067	0.068	0.077	0.040	0.086
**LPC a C28:1**	0.095	0.162	0.102	0.124	0.090	0.153	0.094	0.062	0.061	0.078	0.083	0.072	0.190	0.063	0.103	0.106	0.101	0.119	0.077	0.099
**PC aa C28:1**	1.389	2.372	1.758	1.827	0.869	2.359	1.825	0.900	0.816	1.934	1.365	1.117	2.762	1.332	1.512	1.095	2.396	1.671	1.171	2.178
**PC aa C30:0**	2.389	3.149	2.706	3.148	1.256	3.640	5.623	1.167	1.693	3.472	2.373	2.814	2.980	2.887	2.058	1.453	3.100	2.226	3.411	2.643
**PC aa C30:2**	0.005	0.005	0.099	0.108	0.028	0.176	0.005	0.033	0.165	0.230	0.008	0.140	0.005	0.005	0.005	0.020	0.168	0.005	0.005	0.005
**PC aa C32:0**	125.63	145.25	152.50	201.59	77.721	339.95	366.3	83.603	103.32	189.38	155.58	235.21	97.08	134.58	139.87	104.56	263.13	145.31	217.59	195.19
**PC aa C32:1**	175.49	387.71	243.72	264.33	101.97	557.74	533.68	73.621	70.221	429.29	323.01	154.92	219.93	182.03	132.69	134.79	465.12	209.55	250.07	172.21
**PC aa C32:2**	18.334	22.073	19.708	19.061	12.173	37.801	32.218	6.500	12.095	39.112	23.987	9.939	30.387	16.521	11.947	18.256	22.541	27.910	12.636	19.743
**PC aa C32:3**	1.673	2.732	2.250	2.391	1.479	4.049	3.210	1.984	1.012	3.571	1.484	1.075	2.983	1.737	2.629	2.030	1.827	2.228	0.964	1.986
**PC aa C34:1**	1584.8	2322.5	1804.6	2344.6	1056.3	4074.2	2217.3	1087.6	600.8	2545.8	2812.2	1013.5	1447.9	1164.4	1388.1	1624.9	2375.4	1775	1409.2	1661.9
**PC aa C34:2**	2349.6	3045.5	1704.7	3347.7	2149.6	4671.1	3375.5	1433.5	928.1	3276.5	3433.3	931.9	2392.6	1389.3	1287.6	2117	2797.5	3341.8	1325.1	2298
**PC aa C34:3**	51.173	98.632	48.719	75.752	33.495	120.78	83.246	38.054	23.517	100.05	64.923	26.346	74.940	47.897	47.652	65.334	64.763	83.148	38.526	48.659
**PC aa C34:4**	3.307	5.312	4.037	2.872	2.095	4.766	3.737	2.406	2.708	5.947	2.938	1.645	9.618	2.826	2.782	3.562	4.398	5.129	1.938	5.721
**PC aa C36:0**	1.486	4.641	5.288	7.027	2.357	4.430	5.752	4.437	4.251	6.877	4.446	5.199	5.451	2.891	4.766	3.062	6.544	8.008	1.715	4.045
**PC aa C36:1**	121.6	154.6	139.3	232.01	52.396	335.15	186.60	85.354	69.877	178.84	158.00	117.35	119.84	98.012	147.02	140.12	188.34	112.81	128.58	75.463
**PC aa C36:2**	603.85	721.80	584.86	1265.3	399.78	1459.6	1136.4	454.66	411.66	1016.2	855.12	519.71	638.13	405.03	550.31	512.62	804.17	931.95	464.08	401.14
**PC aa C36:3**	370.84	399.26	258.87	354.73	191.27	527.96	444.36	402.46	194.57	473.05	274.33	197.27	349.44	132.2	208.79	198.01	304.45	431.07	204.73	222
**PC aa C36:4**	493.08	850.02	587.42	461.56	378.87	748.64	494.9	391.95	289.18	827.64	480.13	204.40	1022.2	315.94	447.4	343.51	745.61	884.69	198.14	1476.7

**PC aa C36:5**	39.923	61.006	30.019	23.315	13.704	51.738	31.281	24.106	13.360	66.059	20.593	19.447	57.214	20.384	97.671	31.324	34.486	51.571	13.582	47.169
**PC aa C36:6**	1.407	2.466	1.920	1.698	0.795	2.575	1.938	1.173	1.140	2.425	1.245	0.884	3.024	1.474	2.110	1.356	1.717	2.343	0.662	1.682
**PC aa C38:0**	2.226	2.305	2.960	3.885	1.430	4.069	5.301	4.472	3.923	2.923	2.382	6.375	5.042	1.612	4.919	2.572	9.338	4.225	2.258	2.129
**PC aa C38:1**	0.820	0.525	1.216	1.844	1.001	2.315	0.810	0.141	1.098	0.975	0.633	1.025	1.674	0.716	0.490	0.910	1.579	0.757	0.813	0.256
**PC aa C38:3**	68.015	77.979	61.634	74.542	29.689	91.442	83.003	76.352	53.123	70.838	42.068	39.549	82.685	30.194	62.894	31.884	60.488	81.985	40.770	39.979
**PC aa C38:4**	158.71	225.15	222.29	207.45	85.147	272.42	205.39	142.02	149.82	277.66	125.64	110.57	293.81	110.77	190.25	111.68	221.36	309.24	74.429	333.7
**PC aa C38:5**	70.061	85.475	84.263	65.633	32.458	104.18	67.953	72.708	48.878	116.95	39.687	47.965	108.13	34.525	91.693	42.411	96.910	112.49	32.115	118.88
**PC aa C38:6**	124.29	151.25	130.43	107.44	42.870	179.09	164.02	159.75	97.381	165.71	92.206	47.896	201.79	60.326	176.61	85.280	160.48	216.08	48.033	166.68
**PC aa C40:1**	0.483	0.549	0.585	0.556	0.414	0.637	0.630	0.704	0.488	0.823	0.593	0.554	0.555	0.516	0.559	0.462	0.637	0.783	0.637	0.676
**PC aa C40:2**	0.451	0.614	0.749	0.498	0.500	0.616	0.654	0.911	0.457	0.588	0.423	0.769	0.587	0.467	0.532	0.571	0.698	0.618	0.555	0.303
**PC aa C40:3**	0.371	0.667	1.036	0.561	0.355	0.843	0.603	1.106	0.672	0.684	0.292	0.701	0.767	0.294	0.466	0.355	0.553	0.858	0.522	0.434
**PC aa C40:4**	2.958	3.269	4.572	4.441	1.420	4.827	3.809	4.647	4.624	3.622	2.116	3.674	5.610	1.462	2.481	2.008	7.131	4.343	2.457	3.699
**PC aa C40:5**	8.385	10.263	12.545	14.314	2.800	15.016	8.517	8.464	9.158	12.808	4.878	6.765	11.513	4.236	7.303	3.990	13.734	12.040	4.499	12.757
**PC aa C40:6**	26.427	32.802	32.229	34.639	6.075	44.442	47.902	35.123	30.557	36.449	17.527	16.328	37.586	15.448	56.784	20.068	34.306	50.493	12.272	29.166
**PC aa C42:0**	0.151	0.263	0.280	0.357	0.154	0.517	0.439	0.353	0.285	0.483	0.417	0.451	0.482	0.223	0.451	0.300	0.486	0.590	0.170	0.342
**PC aa C42:1**	0.178	0.177	0.217	0.221	0.189	0.459	0.408	0.302	0.247	0.229	0.181	0.255	0.253	0.166	0.188	0.157	0.248	0.269	0.173	0.135
**PC aa C42:2**	0.049	0.206	0.193	0.063	0.139	0.314	0.195	0.226	0.096	0.155	0.211	0.219	0.262	0.079	0.179	0.135	0.147	0.313	0.139	0.186
**PC aa C42:4**	0.153	0.170	0.239	0.130	0.084	0.215	0.268	0.491	0.128	0.272	0.219	0.191	0.162	0.117	0.161	0.093	0.186	0.206	0.109	0.177
**PC aa C42:5**	0.159	0.128	0.226	0.340	0.111	0.266	0.379	0.427	0.393	0.279	0.154	0.334	0.393	0.077	0.247	0.228	0.653	0.288	0.066	0.332
**PC aa C42:6**	0.188	0.237	0.226	0.258	0.273	0.621	0.246	0.465	0.362	0.397	0.266	0.403	0.620	0.221	0.428	0.373	0.533	0.519	0.296	0.380
**PC ae C30:0**	0.208	0.213	0.200	0.231	0.111	0.305	0.366	0.103	0.172	0.261	0.200	0.291	0.282	0.184	0.165	0.174	0.265	0.191	0.247	0.167
**PC ae C30:1**	0.048	0.021	0.068	0.110	0.016	0.190	0.198	0.036	0.137	0.132	0.087	0.088	0.143	0.040	0.016	0.022	0.043	0.165	0.030	0.016
**PC ae C30:2**	0.027	0.050	0.049	0.067	0.037	0.082	0.057	0.037	0.025	0.082	0.040	0.039	0.090	0.041	0.054	0.042	0.061	0.065	0.033	0.046
**PC ae C32:1**	14.280	17.536	16.370	16.047	8.658	44.269	38.957	13.577	19.899	24.500	22.312	22.393	19.308	13.037	12.768	12.146	28.848	20.119	19.215	11.951
**PC ae C32:2**	3.492	4.676	4.615	2.955	3.097	10.287	9.239	3.487	5.021	4.658	4.071	4.992	4.447	2.944	4.092	2.703	4.320	5.110	3.726	3.041
**PC ae C34:0**	6.010	8.938	9.172	11.232	3.288	13.467	13.169	3.831	4.944	8.602	6.521	8.239	7.454	6.799	7.193	4.333	9.530	6.781	11.044	8.493
**PC ae C34:1**	59.345	91.884	57.706	94.780	37.345	161.03	96.857	37.168	31.174	89.234	76.397	72.656	67.511	50.353	58.168	54.251	116.15	64.332	85.099	48.770
**PC ae C34:2**	30.843	39.549	36.643	42.661	27.443	79.058	51.112	23.379	26.421	52.360	37.443	31.261	55.551	26.123	27.954	32.540	41.329	50.719	33.998	26.470
**PC ae C34:3**	11.642	13.078	21.353	11.268	12.379	29.502	32.199	11.010	16.469	18.320	14.850	17.602	32.352	8.764	12.847	8.398	14.292	21.928	13.514	12.399
**PC ae C36:0**	4.992	4.635	5.793	5.768	1.775	7.515	7.033	3.120	3.523	5.868	5.956	2.877	3.632	7.036	4.830	3.720	3.085	5.994	4.265	3.338
**PC ae C36:1**	29.941	43.497	39.038	37.140	27.466	54.561	37.123	28.793	18.660	36.182	30.313	19.698	29.252	23.200	29.011	24.944	28.293	33.901	25.893	26.845
**PC ae C36:2**	43.487	64.015	35.759	52.477	33.030	77.027	50.009	25.175	20.270	58.283	38.212	29.291	42.687	23.138	36.213	34.134	41.595	50.694	32.622	29.728

**PC ae C36:3**	14.084	20.314	17.322	22.522	11.601	34.720	22.569	14.781	14.192	23.113	17.890	19.134	24.602	9.548	13.856	13.165	23.080	25.537	16.286	11.038
**PC ae C36:4**	24.844	23.849	38.104	41.962	21.971	45.786	36.338	35.296	41.119	37.012	29.261	52.329	75.743	16.416	25.940	23.133	117.05	53.210	24.699	41.916
**PC ae C36:5**	16.904	17.619	31.034	14.035	15.546	37.990	25.894	24.506	34.383	23.300	19.552	31.923	47.710	10.697	19.977	14.435	44.546	39.704	16.212	31.474
**PC ae C38:0**	2.358	2.619	2.225	2.290	1.075	2.875	2.633	2.977	1.364	3.277	1.470	1.085	4.119	1.370	2.857	1.712	2.167	3.970	1.137	2.600
**PC ae C38:1**	0.962	1.581	2.576	1.930	2.290	3.165	2.683	3.920	3.042	1.471	1.081	1.831	2.314	0.824	2.221	0.700	1.763	1.801	0.749	0.624
**PC ae C38:2**	3.398	3.793	5.813	4.104	3.602	7.418	4.425	9.470	4.498	3.191	2.567	3.679	4.231	2.388	3.472	2.330	3.586	3.782	3.108	1.618
**PC ae C38:3**	7.427	9.649	15.910	7.321	11.125	11.154	8.716	20.970	10.635	8.633	4.590	5.779	12.360	5.081	7.722	4.186	7.128	9.700	5.051	6.025
**PC ae C38:4**	18.290	24.382	23.727	23.153	12.506	26.568	19.971	21.008	21.815	26.491	16.909	21.605	35.245	11.415	18.894	14.348	39.869	29.861	14.514	31.591
**PC ae C38:5**	18.666	17.771	30.943	26.187	17.462	38.287	24.921	43.366	36.651	26.705	20.822	53.775	47.643	11.568	29.397	18.318	81.208	39.133	22.989	25.559
**PC ae C38:6**	6.289	6.977	10.131	10.361	4.427	14.132	12.927	10.553	11.348	10.549	6.521	13.899	17.990	5.176	12.950	6.433	18.704	14.710	5.726	9.082
**PC ae C40:1**	0.372	0.668	1.116	0.413	0.600	1.076	0.702	2.090	1.294	1.069	0.466	0.897	1.354	0.268	0.611	0.718	0.683	1.270	0.224	0.911
**PC ae C40:2**	1.462	1.823	1.760	1.540	1.467	1.922	2.044	2.920	1.353	1.576	1.397	1.162	2.108	1.054	2.467	1.355	1.032	1.775	1.088	1.699
**PC ae C40:3**	1.215	1.315	4.203	1.456	2.233	2.616	1.883	6.073	4.185	1.337	0.851	1.119	2.358	0.908	1.764	0.839	1.294	1.494	1.053	1.297
**PC ae C40:4**	1.633	1.730	3.998	2.296	1.748	3.000	2.259	5.218	3.742	2.479	1.925	2.597	4.118	1.363	2.013	1.980	3.277	3.357	1.595	2.621
**PC ae C40:5**	2.802	2.709	7.592	3.927	3.081	5.564	3.743	14.475	7.973	3.662	2.883	5.829	7.389	1.576	4.099	2.043	7.971	5.360	2.907	3.349
**PC ae C40:6**	3.953	4.262	4.566	5.080	1.558	6.282	5.172	5.118	4.665	4.614	2.439	5.469	6.248	2.615	7.153	3.116	9.964	7.388	2.695	4.177
**PC ae C42:0**	0.680	0.808	1.056	0.778	0.749	1.148	0.873	1.040	0.795	0.842	0.977	0.796	0.742	0.743	0.776	0.596	0.842	0.978	0.566	0.748
**PC ae C42:1**	0.381	0.533	0.773	0.519	0.344	0.647	0.516	0.762	0.556	0.499	0.353	0.531	0.686	0.251	0.504	0.354	0.581	0.712	0.341	0.546
**PC ae C42:2**	0.213	0.305	0.384	0.264	0.175	0.534	0.439	0.485	0.392	0.478	0.293	0.301	0.489	0.185	0.571	0.142	0.253	0.283	0.262	0.191
**PC ae C42:3**	0.288	0.258	0.573	0.357	0.531	0.691	0.479	1.689	0.797	0.693	0.495	0.442	0.651	0.296	0.488	0.323	0.551	0.704	0.276	0.690
**PC ae C42:4**	0.379	0.336	0.607	0.367	0.183	0.738	0.272	0.504	0.721	0.261	0.268	0.588	0.524	0.278	0.351	0.211	0.461	0.677	0.252	0.305
**PC ae C42:5**	1.138	0.618	1.954	0.982	0.695	1.685	1.364	3.349	2.265	1.146	0.902	1.959	2.178	0.834	1.353	0.953	1.818	1.839	0.974	1.216
**PC ae C44:3**	0.086	0.066	0.249	0.128	0.116	0.107	0.212	0.382	0.108	0.098	0.096	0.116	0.202	0.105	0.091	0.058	0.240	0.178	0.115	0.061
**PC ae C44:4**	0.287	0.106	0.148	0.052	0.089	0.221	0.121	0.423	0.187	0.153	0.171	0.123	0.168	0.086	0.195	0.098	0.140	0.148	0.093	0.184
**PC ae C44:5**	0.422	0.312	0.389	0.419	0.270	0.710	0.573	0.587	0.752	0.416	0.468	0.771	0.755	0.277	0.459	0.312	0.685	0.661	0.379	0.348
**PC ae C44:6**	0.298	0.310	0.408	0.271	0.186	0.803	0.462	0.478	0.709	0.418	0.289	0.637	0.579	0.251	0.520	0.266	0.577	1.041	0.403	0.247
**Sugars**	4430.6	3860.5	5173.6	5106.3	7982.6	5473.1	5216.2	7194.9	7265.3	6874.3	8156.6	6474.9	7770.9	4941.7	8043.4	6111.5	10401	6787.8	11334	8207.7
**Ala**	165	80.7	604	353	132	207	1080	405	579	213	217	713	193	171	351	208	304	214	908	527
**Arg**	73	36.5	8.2	77.3	51.8	37.3	179	98.7	26	62.4	60.7	155	76	28.7	132	38.3	112	79.7	343	62.4
**Asn**	45.2	30.8	2.11	60.8	27.9	41.6	190	74.6	104	44	4.67	194	36.3	19.6	32.1	47.1	77.8	51.4	78.7	50.2
**Asp**	7.7	10.9	11.4	9.22	12.2	13.9	50.1	14.5	30.7	8	34.1	15.5	11.3	7.96	13.9	5.63	6.19	5.98	44.3	24.4
**Cit**	17.2	3.67	8.38	12.3	3.42	10.8	33	29.1	40.5	3.92	5.06	55.4	5.07	5.12	4.58	7.64	15.2	9.52	45.8	3.92

**Gln**	716	293	427	822	22.1	775	2390	853	544	429	283	2050	389	156	429	463	1230	371	1580	413
**Glu**	31.1	21.7	163	16.8	250	61.8	146	109	846	22.5	74.9	69	150	27.6	159	20	99.2	15.7	78.2	36.2
**Gly**	155	83.9	250	171	156	212	722	245	575	135	147	888	182	148	368	136	183	138	868	204
**His**	105	56.6	120	170	54.3	97.6	353	112	139	110	86.2	225	145	79.1	134	99.1	115	70.4	213	98.2
**Ile**	62.6	36.2	57.9	70.2	62.4	52.4	145	151	190	58.1	122	76.4	58.9	30	98.4	42.1	92.1	73.6	184	48.6
**Leu**	130	77	129	135	120	116	267	280	316	133	235	123	93.3	68.6	156	99.6	204	159	318	126
**Lys**	291	160	277	411	212	131	764	472	1060	237	261	766	215	114	300	158	311	257	613	276
**Met**	14.7	13.4	45.9	37.8	20.1	25.9	175	56.4	170	23.9	23	392	12.6	12.2	46.1	19.1	35.3	20.6	204	40.9
**Orn**	83.3	27	155	63.1	40.8	57	237	126	485	50.2	64.5	166	41.9	31.5	214	24.3	70.8	79.3	344	74.1
**Phe**	105	64	206	129	101	112	339	139	386	213	128	289	166	289	236	119	178	103	442	113
**Pro**	114	50.7	169	228	92	128	14	271	554	110	75.2	1050	87	47.4	201	61.3	369	78.5	704	114
**Ser**	37.8	38.3	64.3	35.2	82.3	46	161	155	145	34.1	53	100	35.8	32.7	116	33	73.9	37.1	190	71.7
**Thr**	50	41.1	120	76.8	52.6	55.3	3	180	236	62.3	54.5	335	73.6	39	110	52.4	99.3	77.3	213	105
**Trp**	19.5	14.9	55.8	16.5	29.1	10.7	45.1	114	195	26.5	24.5	40.4	10.8	8.320	82.4	9.490	13.7	27.9	76	47.2
**Tyr**	50.2	29.7	97.1	72.7	79.8	77.1	179	187	343	135	46.5	239	43	25.3	65.6	47.5	138	60.2	159	87.6
**Val**	189	123	229	222	199	181	425	531	559	261	518	260	207	141	403	224	366	333	941	256
**Ac Orn**	0.672	0.086	0.919	0.378	0.105	0.339	1.470	0.367	0.689	0.559	0.442	1.260	0.168	0.240	0.635	0.250	0.387	0.568	0.513	0.305
**ADMA**	0.503	0.147	0.210	0.436	0.402	0.542	1.290	0.633	2.770	0.800	0.411	2.400	0.561	0.332	0.528	0.485	1.820	0.591	1.950	0.437
**Alpha AAA**	1.510	0.564	3.130	1.120	1.410	1.480	7.630	1.520	5.460	0.708	0.673	1.900	1.030	0.719	0.924	1.410	0.545	1.420	1.180	0.896
**Creatinine**	230	38.200	329	142	51.8	231	250	87.5	172	274	194	315	418	276	215	114	140	289	117	118
**DOPA**	0.220	0.040	0.442	0.146	0.040	0.131	0.492	0.206	0.342	0.173	0.105	0.338	0.040	0.114	0.203	0.118	0.218	0.136	0.481	0.295
**Kynurenine**	5.880	2.390	19	8.660	5.680	7.440	13.8	5.8	20.3	10.2	8.730	13.4	27	21.3	12.3	13.5	21.2	12.7	14.4	7.51
**Met SO**	1.070	0.605	8.360	1.780	1.420	2.060	9.170	2.060	17.700	1.390	1.870	10.400	3.030	2.030	5.080	0.631	3.530	1.550	6.900	1.430
**Putrescine**	0.089	0.100	0.781	0.152	0.285	0.015	0.708	0.015	0.015	0.148	0.289	0.519	0.146	0.109	1.360	0.015	0.146	0.128	0.175	0.335
**SDMA**	1.880	0.541	2.330	1.070	0.150	1.470	1.550	0.996	2.490	1.890	1.270	3.620	1.170	1.560	3.570	0.708	1.920	3.220	1.590	0.652
**Spermidine**	0.076	0.126	0.110	0.031	0.058	0.035	0.203	0.072	0.116	0.047	0.038	0.076	0.041	0.037	0.103	0.107	0.031	0.045	0.109	0.104
**Spermine**	0.060	0.090	0.049	0.018	0.023	0.058	0.122	0.028	0.027	0.044	0.029	0.032	0.043	0.017	0.020	0.037	0.024	0.034	0.064	0.027
**T4 OH Pro**	5.620	2.320	7.610	13.2	20.4	15.4	97.8	8.9	58.9	7.970	17.4	118	10.8	5.08	5.41	6.09	15.1	17.5	32	13
**Taurine**	45.2	32.5	236	37.3	29.6	89.8	302	50.1	54.2	11.9	9.41	17.7	15.9	12.3	23.8	16.1	11.3	13.4	44	84.3
**Total DMA**	2.340	0.579	2.790	1.380	0.788	1.610	2.000	1.080	4.080	2.480	1.500	5.660	1.260	1.720	3.290	0.902	2.930	3.180	2.600	1.050
**SM OH C14:1**	1.927	2.896	2.265	2.783	1.496	3.068	2.190	1.383	0.899	2.222	1.842	1.350	3.277	1.898	2.451	1.530	3.296	2.218	1.772	2.466
**SM OH C16:1**	0.879	0.958	0.833	0.990	0.674	1.138	0.736	0.709	0.415	0.744	0.521	0.414	1.207	0.611	1.026	0.536	0.893	0.722	0.482	1.002

**SM OH C22:1**	0.317	0.375	0.571	0.426	0.225	0.444	0.399	0.473	0.315	0.382	0.330	0.210	0.930	0.380	0.432	0.297	0.388	0.521	0.259	0.471
**SM OH C22:2**	0.323	0.289	0.494	0.452	0.217	0.454	0.434	0.421	0.313	0.445	0.384	0.232	0.938	0.283	0.638	0.309	0.391	0.626	0.248	0.506
**SM OH C24:1**	0.024	0.006	0.036	0.031	0.017	0.044	0.053	0.036	0.025	0.033	0.017	0.018	0.061	0.021	0.053	0.012	0.035	0.033	0.052	0.023
**SM C16:0**	26.732	35.687	37.148	38.679	31.029	57.247	38.603	41.224	28.152	33.262	31.637	27.446	51.885	27.100	41.391	23.353	92.879	37.212	28.322	32.106
**SM C16:1**	3.268	5.583	5.135	6.245	4.346	9.005	6.496	6.564	3.909	7.046	5.535	4.926	9.491	4.257	7.438	3.720	11.318	6.502	3.235	5.237
**SM C18:0**	5.202	5.229	5.850	4.941	5.374	8.193	5.590	6.449	2.552	4.407	3.413	1.540	7.013	2.924	5.871	2.588	5.014	4.092	2.598	6.146
**SM C18:1**	1.819	2.395	2.654	2.579	2.134	4.023	2.357	3.419	1.221	3.175	1.856	0.862	3.773	1.317	3.600	1.345	2.242	2.521	0.880	2.856
**SM C20:2**	0.068	0.117	0.067	0.113	0.055	0.132	0.136	0.096	0.058	0.095	0.046	0.041	0.071	0.063	0.161	0.044	0.080	0.123	0.036	0.074
**SM C22:3**	0.012	0.005	0.045	0.118	0.059	0.074	0.058	0.036	0.035	0.005	0.005	0.005	0.005	0.023	0.005	0.025	0.099	0.036	0.005	0.014
**SM C24:0**	0.354	0.531	0.810	0.539	0.396	0.572	0.614	0.742	0.551	0.604	0.471	0.437	1.188	0.396	0.599	0.292	0.611	0.796	0.460	0.572
**SM C24:1**	1.303	1.356	2.197	1.577	2.483	2.490	2.052	2.997	1.640	1.522	1.865	1.513	2.939	1.313	3.382	1.129	2.434	2.133	1.650	1.740
**SM C26:1**	0.008	0.002	0.002	0.005	0.016	0.014	0.013	0.003	0.007	0.005	0.010	0.008	0.014	0.006	0.019	0.008	0.016	0.002	0.002	0.009
**C0**	36.538	8.516	91.795	24.626	37.951	55.467	90.904	55.998	91.804	30.205	50.097	71.447	27.299	26.090	66.774	34.947	58.050	32.785	75.781	25.816
**C2**	7.226	1.974	11.066	9.233	4.046	25.234	39.580	3.337	9.086	17.874	16.299	33.723	3.021	3.895	5.080	6.099	20.724	7.839	8.886	3.464
**C4**	0.330	0.173	0.789	0.364	0.271	0.676	0.878	0.325	0.239	0.336	0.740	0.424	0.222	0.178	0.507	0.200	0.660	0.574	0.720	0.180

Table S2 – Plasma concentration (µM) of quantified metabolites for each patient at day 7 and survival rate at 28 and 90 days (S, survivor; NS, non survivor).

Metabolite D7	#1	#2	#3	#4	#5	#6	#7	#8	#9	#10	#11	#12	#13	#14	#15	#16	#17	#18	#19	#20
28 day survival	S	S	NS	NS	NS	NS	S	S	S	S	NS	NS	S	S	S	NS	NS	S	NS	S
90 day survival	NS	S	NS	NS	NS	NS	S	NS	S	S	NS	NS	S	S	S	NS	NS	S	NS	S
LPC a C16:1	2.750	2.507	1.121	0.576	1.550	4.000	2.369	0.494	2.327	3.721	1.741	1.256	3.004	1.378	2.926	0.936	0.762	2.258	0.630	0.858
LPC a C17:0	1.210	1.013	0.340	0.269	0.339	0.850	0.787	0.131	0.508	1.006	0.488	0.279	0.697	0.222	0.709	0.534	0.293	1.122	0.130	0.442
LPC a C18:0	12.317	9.893	4.924	5.684	6.594	10.404	10.807	3.342	8.825	15.960	7.721	3.080	14.744	2.346	11.795	6.908	3.642	17.854	1.409	5.309
LPC a C18:1	17.099	20.421	10.772	7.772	12.801	30.973	13.072	5.807	16.064	27.513	12.215	7.810	10.495	3.109	14.485	15.905	4.872	19.699	5.000	5.600
LPC a C18:2	10.566	19.960	8.192	6.823	7.092	22.995	8.158	5.064	17.805	15.080	3.789	5.868	5.436	1.683	7.052	21.953	3.256	11.406	5.355	5.641
LPC a C20:3	1.493	1.338	0.872	0.406	1.316	3.745	1.235	0.267	0.746	2.113	0.955	0.367	0.999	0.382	1.008	0.989	0.490	1.424	0.612	0.486
LPC a C20:4	3.671	2.916	1.606	0.978	2.973	5.801	2.011	0.667	2.068	4.438	1.240	0.967	2.873	0.898	2.059	2.404	0.807	3.307	0.585	1.515
LPC a C24:0	0.093	0.104	0.080	0.085	0.066	0.096	0.142	0.075	0.102	0.093	0.060	0.086	0.096	0.064	0.109	0.062	0.066	0.120	0.041	0.084
LPC a C28:1	0.143	0.141	0.080	0.123	0.076	0.110	0.135	0.085	0.063	0.122	0.090	0.160	0.203	0.149	0.139	0.070	0.116	0.110	0.072	0.138
PC aa C28:1	1.907	2.100	1.487	1.955	0.847	1.709	1.579	1.118	1.183	2.330	1.567	2.736	2.781	1.852	1.746	1.435	2.332	1.635	0.748	1.794
PC aa C30:0	3.132	4.081	2.850	4.462	1.871	3.080	4.676	2.781	3.208	3.987	3.277	7.661	5.166	4.296	3.135	1.493	4.093	2.901	2.959	4.051
PC aa C30:2	0.005	0.005	0.037	0.100	0.005	0.059	0.070	0.060	0.074	0.005	0.274	0.356	0.005	0.215	0.247	0.047	0.122	0.005	0.083	0.119
PC aa C32:0	168.78	187.16	142.05	331.74	96.986	168.03	268.98	207.7	164.15	173.83	188.01	451.81	201.3	134.24	154.36	132.2	404.2	170.8	188.16	206.51
PC aa C32:1	249.77	266.14	194.71	287.7	144.29	416.55	395.58	174.32	162.21	268.43	343.82	435.23	382.44	572.41	389.91	101.31	774.17	159.55	290.92	230.62
PC aa C32:2	23.973	49.502	11.919	24.009	12.084	42.205	43.367	17.235	15.876	22.052	17.212	28.395	40.072	53.781	25.310	16.069	39.420	30.674	13.696	36.692
PC aa C32:3	2.694	4.149	1.432	2.603	2.206	3.896	4.271	1.442	1.310	3.482	1.932	1.965	5.785	5.123	3.486	2.447	2.019	3.830	1.049	2.992
PC aa C34:1	2036	2314.4	1562.4	3559.7	1233.6	2870.3	2695.1	2038.2	865.9	2356.5	2990.5	1478.8	2322.9	1353.2	2319.5	2022.3	3740.3	1923.7	1160.9	2088.5
PC aa C34:2	2141.4	3034.9	1229.9	4325.2	781.9	2300.1	2686.5	2668.1	1327.7	1823.5	1877.5	1350.2	1899. 2	1187.6	1077	3458.1	3832.7	2564.1	1227.1	3205.1
PC aa C34:3	77.561	172.34	40.878	130.95	51.929	129.92	141.35	55.915	43.644	107.79	69.660	56.852	152.12	143.65	108.58	93.371	135.10	115.73	54.052	127.87
PC aa C34:4	6.528	12.192	2.446	5.464	4.618	11.793	9.174	2.801	2.849	7.929	3.804	4.150	14.307	12.474	8.734	3.944	6.489	8.492	2.341	8.604
PC aa C36:0	5.274	7.325	3.387	6.533	1.593	7.179	8.500	5.568	6.927	4.892	1.481	7.186	6.045	3.309	2.284	5.637	9.316	5.215	2.033	7.056
PC aa C36:1	148.52	167.98	182.47	478.68	154.35	347.03	268.44	224.96	89.821	230.5	248.23	169.76	342.52	134.22	468.42	196.72	268.91	193.79	91.290	231.59
PC aa C36:2	598.4	910.25	636.61	1763.2	340.08	837.16	845.83	899.11	525.52	761.24	664.54	674.84	661.02	334.49	643.28	973.42	1089.6	949.81	426.31	990.12
PC aa C36:3	496.14	781.71	287.62	513.29	277.40	508.53	512.38	260.44	289.29	563.62	548.31	383.83	501.06	304.02	373.16	433.00	602.14	663.95	407.22	380.54
PC aa C36:4	615.67	792.36	268.09	426.30	369.82	642.19	508.83	244.08	200.10	694.66	400.26	364.54	994.50	480.46	510.86	368.73	574.95	660.61	196.54	724.87
PC aa C36:5	52.972	84.406	25.797	28.331	32.121	135.12	52.253	18.540	23.250	117.47	21.721	36.503	105.54	77.650	113.74	48.427	39.509	66.158	19.275	48.539

**PC aa C36:6**	2.366	4.228	1.283	2.445	1.059	4.844	3.110	1.443	1.233	3.719	1.467	2.211	3.881	2.639	3.473	1.796	2.077	3.006	1.068	2.301
**PC aa C38:0**	5.829	3.538	2.781	5.435	1.896	7.118	9.661	4.170	4.567	4.847	3.715	10.055	4.631	3.387	6.338	4.617	10.230	7.088	2.121	4.277
**PC aa C38:1**	0.990	2.180	1.734	0.374	0.504	1.989	2.531	1.762	2.211	2.446	0.069	0.388	1.021	0.658	0.843	1.365	1.133	3.499	0.156	0.000
**PC aa C38:3**	90.577	104.07	59.051	87.135	84.848	115.97	109.53	38.895	47.935	111.18	101.82	71.647	151.6	71.059	127.39	46.071	82.570	115.50	50.772	74.177
**PC aa C38:4**	198.27	188.16	114.50	239.94	168.95	230.44	182.81	108.59	79.353	236.87	130.6	132.15	435.24	132.73	275.75	128.19	207.47	236.56	59.148	252.15
**PC aa C38:5**	116.96	118.82	44.280	76.642	64.701	143.42	84.406	36.125	38.712	137.42	48.996	46.907	185.41	53.837	132.48	54.638	104.12	132.02	27.424	90.264
**PC aa C38:6**	155.16	102.69	43.300	105.39	46.501	138.73	153.13	57.988	43.902	151.82	55.175	32.700	125.8	44.638	136.40	109.61	145.03	170.24	21.440	112.62
**PC aa C40:1**	0.675	0.601	0.543	0.783	0.474	0.536	0.935	0.676	0.726	0.669	0.563	0.752	0.671	0.722	0.681	0.422	0.834	0.664	0.486	0.780
**PC aa C40:2**	0.544	0.900	0.689	0.779	0.703	0.607	1.112	0.648	0.749	0.761	0.688	1.086	0.764	0.549	0.502	0.555	0.896	0.862	0.604	0.568
**PC aa C40:3**	0.698	0.839	0.846	1.353	0.573	0.719	1.461	0.599	0.714	0.874	0.830	1.360	1.290	0.568	0.601	0.746	1.765	1.224	0.809	0.897
**PC aa C40:4**	4.987	4.058	2.774	5.669	3.484	5.305	4.817	2.928	3.207	3.570	3.458	4.029	8.076	2.580	4.932	2.473	8.854	5.846	2.183	5.720
**PC aa C40:5**	12.230	8.047	5.240	13.712	6.644	14.921	10.595	5.515	5.005	11.011	4.466	6.140	18.502	5.225	13.741	6.375	15.472	14.081	3.757	13.377
**PC aa C40:6**	30.574	17.710	13.348	32.010	15.010	33.292	37.375	12.873	10.530	34.100	12.467	9.643	35.919	10.088	57.616	21.901	32.447	40.677	5.819	26.914
**PC aa C42:0**	0.414	0.431	0.226	0.564	0.156	0.645	0.778	0.232	0.439	0.450	0.260	0.574	0.493	0.445	0.449	0.415	0.552	0.787	0.246	0.447
**PC aa C42:1**	0.344	0.269	0.189	0.266	0.245	0.250	0.342	0.151	0.219	0.141	0.107	0.279	0.468	0.340	0.345	0.239	0.422	0.408	0.198	0.419
**PC aa C42:2**	0.194	0.222	0.177	0.238	0.123	0.213	0.286	0.302	0.323	0.153	0.215	0.371	0.278	0.148	0.205	0.291	0.227	0.267	0.185	0.207
**PC aa C42:4**	0.107	0.187	0.237	0.369	0.082	0.413	0.192	0.109	0.214	0.149	0.189	0.331	0.343	0.115	0.282	0.226	0.312	0.204	0.112	0.254
**PC aa C42:5**	0.314	0.336	0.233	0.316	0.238	0.186	0.250	0.301	0.175	0.305	0.183	0.500	0.413	0.174	0.312	0.304	0.570	0.316	0.245	0.451
**PC aa C42:6**	0.536	0.382	0.334	0.702	0.297	0.479	0.518	0.249	0.518	0.421	0.476	0.658	0.617	0.344	0.447	0.388	0.721	0.365	0.265	0.292
**PC ae C30:0**	0.283	0.301	0.251	0.475	0.130	0.254	0.417	0.173	0.251	0.374	0.276	0.907	0.288	0.366	0.220	0.147	0.312	0.293	0.214	0.323
**PC ae C30:1**	0.046	0.129	0.122	0.090	0.016	0.117	0.179	0.048	0.167	0.077	0.094	0.277	0.128	0.124	0.164	0.068	0.069	0.107	0.118	0.051
**PC ae C30:2**	0.064	0.069	0.044	0.070	0.032	0.077	0.077	0.045	0.035	0.073	0.050	0.088	0.077	0.054	0.071	0.053	0.082	0.064	0.026	0.058
**PC ae C32:1**	27.758	31.071	22.107	40.812	10.006	46.048	69.749	25.331	35.333	29.846	31.845	78.824	26.744	39.233	27.023	19.280	34.558	34.076	18.693	32.126
**PC ae C32:2**	6.148	7.687	5.637	5.919	2.707	10.543	10.880	4.654	7.867	6.342	5.572	14.615	8.596	7.878	6.659	3.877	5.099	7.789	3.720	4.859
**PC ae C34:0**	11.635	11.113	9.573	15.966	3.332	8.397	14.838	6.276	6.081	10.346	7.687	19.726	9.245	7.126	6.883	6.308	12.721	7.204	8.096	8.494
**PC ae C34:1**	102.18	94.683	66.171	144.63	47.508	128.69	150.75	91.301	58.008	99.144	108.74	161.01	91.964	98.187	99.800	66.571	151.39	91.570	75.032	77.242
**PC ae C34:2**	51.199	67.791	37.244	92.822	24.817	81.084	113.9	55.247	45.272	44.946	45.619	64.065	66.512	56.235	43.325	59.188	52.748	78.248	30.923	71.862
**PC ae C34:3**	20.539	28.189	15.776	19.233	12.902	34.449	42.278	20.304	26.979	16.739	18.108	29.641	43.992	27.981	17.480	18.013	16.625	32.086	10.666	20.068
**PC ae C36:0**	5.861	2.832	11.438	6.669	1.231	4.604	7.701	5.700	4.183	4.902	5.125	7.998	5.023	2.851	5.145	3.195	7.829	4.895	3.038	3.227
**PC ae C36:1**	39.346	46.451	37.504	50.540	18.046	51.228	53.240	35.814	21.423	37.752	32.784	36.172	36.215	25.897	47.728	30.269	41.492	34.413	19.077	35.152
**PC ae C36:2**	56.369	72.835	36.010	99.458	21.442	65.848	70.978	48.006	30.824	46.104	42.593	48.540	43.900	33.955	40.995	62.851	57.628	61.528	28.111	69.204
**PC ae C36:3**	28.730	32.186	17.286	42.817	14.228	40.225	59.529	34.816	21.126	27.248	30.503	27.852	31.531	30.411	25.208	31.540	32.656	43.501	18.177	26.169

**PC ae C36:4**	59.126	50.398	31.416	57.318	26.812	79.505	90.168	32.668	49.851	54.051	59.660	98.348	73.540	56.323	39.445	42.869	122.66	69.836	22.559	62.643
**PC ae C36:5**	40.009	38.045	24.566	20.751	17.208	65.108	46.751	19.331	46.209	28.810	23.337	53.097	46.500	40.898	25.442	24.236	50.443	52.237	13.609	38.827
**PC ae C38:0**	3.367	3.935	1.678	2.502	1.453	5.208	3.935	1.844	1.854	4.525	1.545	2.226	5.007	1.949	4.497	2.537	3.570	4.221	1.070	2.948
**PC ae C38:1**	1.364	1.778	3.907	3.183	1.487	2.947	3.543	5.329	2.542	1.263	1.734	3.153	1.754	1.573	2.049	1.553	2.663	2.610	1.117	0.911
**PC ae C38:2**	4.806	5.093	5.427	10.588	4.486	4.040	9.471	6.381	5.141	5.730	4.401	6.864	4.485	3.719	4.668	4.813	6.023	5.303	3.588	4.539
**PC ae C38:3**	11.448	12.501	10.770	11.813	9.606	12.678	19.965	9.020	9.312	12.499	10.032	9.492	11.620	10.706	11.580	8.094	8.552	13.361	5.636	8.251
**PC ae C38:4**	35.248	28.867	16.737	34.930	17.102	38.700	47.214	18.186	21.616	32.925	29.200	40.174	41.221	27.383	27.288	20.256	42.606	38.538	12.449	40.442
**PC ae C38:5**	59.291	41.913	27.454	47.061	30.036	70.104	74.883	33.758	53.642	46.380	47.890	99.991	52.357	41.273	43.650	41.137	97.221	67.743	23.467	42.471
**PC ae C38:6**	15.864	12.001	8.180	13.188	6.314	26.676	24.308	8.783	14.374	13.990	9.198	20.521	16.248	12.503	15.169	11.101	22.423	20.199	4.858	11.970
**PC ae C40:1**	1.337	1.836	0.643	0.836	0.844	1.559	1.695	1.101	1.404	1.669	0.812	1.210	1.795	0.660	1.710	1.031	1.288	1.633	0.585	1.166
**PC ae C40:2**	2.237	2.253	2.052	2.984	1.818	2.275	2.691	1.694	2.096	1.907	2.068	1.967	2.041	1.565	2.358	2.591	1.904	2.306	1.136	2.323
**PC ae C40:3**	1.884	1.631	3.211	1.901	2.566	2.315	4.784	3.206	2.746	2.026	1.925	1.820	2.355	2.119	2.259	1.170	1.898	2.524	1.160	2.023
**PC ae C40:4**	3.925	2.868	3.024	4.519	3.143	4.220	6.654	3.392	4.277	3.294	3.066	4.029	5.585	2.565	3.245	3.145	3.425	4.337	1.824	3.887
**PC ae C40:5**	6.674	4.457	4.444	6.877	4.109	8.650	11.727	6.461	6.929	6.300	5.472	11.282	8.114	4.467	6.447	4.328	9.410	10.243	2.422	6.705
**PC ae C40:6**	7.866	4.652	2.991	7.274	3.208	10.451	11.139	4.125	5.867	6.673	4.232	9.809	7.829	5.161	8.243	6.216	11.143	9.723	2.221	7.228
**PC ae C42:0**	0.910	0.779	0.809	0.893	0.697	0.953	1.084	0.685	0.619	0.895	0.652	0.967	0.853	0.585	0.782	0.669	1.191	1.154	0.750	0.731
**PC ae C42:1**	0.542	0.681	0.643	0.594	0.503	0.845	0.560	0.662	0.534	0.547	0.402	0.652	0.790	0.479	0.571	0.439	0.975	0.711	0.359	0.843
**PC ae C42:2**	0.450	0.408	0.275	0.277	0.233	0.352	0.827	0.390	0.276	0.495	0.340	0.611	0.458	0.300	0.523	0.320	0.501	0.393	0.226	0.485
**PC ae C42:3**	0.841	0.834	0.434	0.541	0.454	0.925	1.366	0.640	0.713	0.923	0.648	0.660	0.772	0.373	0.852	0.681	0.700	0.886	0.336	0.602
**PC ae C42:4**	0.826	0.408	0.498	0.650	0.455	0.913	1.439	0.773	1.127	0.594	0.600	0.669	0.749	0.656	0.914	0.661	0.608	0.828	0.241	0.788
**PC ae C42:5**	2.164	1.735	1.256	1.663	1.050	2.752	3.865	1.644	2.764	1.951	2.138	3.318	2.108	1.831	2.666	1.542	1.731	3.711	0.933	1.900
**PC ae C44:3**	0.171	0.133	0.054	0.202	0.141	0.180	0.152	0.131	0.126	0.120	0.117	0.141	0.157	0.108	0.060	0.158	0.166	0.174	0.122	0.076
**PC ae C44:4**	0.164	0.237	0.143	0.249	0.164	0.327	0.274	0.197	0.193	0.297	0.324	0.204	0.219	0.216	0.257	0.225	0.206	0.238	0.168	0.155
**PC ae C44:5**	0.974	0.574	0.411	0.411	0.277	1.733	1.444	0.537	1.160	0.701	0.704	1.523	0.782	0.598	0.707	0.815	0.694	0.858	0.436	0.720
**PC ae C44:6**	0.636	0.626	0.236	0.433	0.346	1.220	1.042	0.393	0.947	0.689	0.519	1.288	0.785	0.766	0.923	0.452	0.804	0.883	0.401	0.884
**Sugars**	8079.7	7240.2	4793.8	4408.4	7463.5	7087.4	8615.3	16726	5410.5	8425.9	8411.7	9153.4	7390.9	10431	7747.9	7737.1	6909	7399.8	11244	13247
**Ala**	228	230	360	484	139	484	527	696	573	325	272	1980	301	339	383	141	282	129	693	288
**Arg**	123	67.6	96.8	101	143	150	122	241	238	115	131	365	165	230	191	76.8	143	90.4	129	58.4
**Asn**	64.4	78	36.3	91.7	18.4	66.4	98.4	80.4	109	93.6	35.7	404	71.8	57.8	33.6	47	87.1	40.5	138	54
**Asp**	4.7	14.7	37.6	21.9	37	5.720	21.7	16.3	10.2	7.340	8.660	16.6	13	9.410	2.760	7.050	15.8	4.490	25.7	22.1
**Cit**	28.4	13.8	22.9	24	9.210	15.5	19	35	15.9	19.2	24.1	215	31.4	17.4	9.950	13	19.6	30.6	40.9	14.7
**Gln**	842	629	38.1	835	24.2	941	589	72.8	423	863	490	3120	516	492	742	650	787	517	1160	637

**Glu**	45.8	107	477	65.1	310	46.5	272	594	307	67.3	152	67.8	349	268	55.2	57	262	98.6	97.1	85.9
**Gly**	237	287	253	303	183	385	380	520	327	274	341	1830	202	473	525	157	297	187	344	216
**His**	73.2	62	118	154	62.8	118	126	109	122	96	75.2	235	124	115	108	53.8	109	60.5	169	72.5
**Ile**	102	52.8	55.1	94.3	98.3	76	80.1	88.5	130	76.2	73.1	202	107	87	111	62.7	52.4	49.1	137	64.8
**Leu**	221	116	108	144	123	147	170	178	226	173	107	358	222	163	160	123	113	114	287	139
**Lys**	365	328	287	344	318	430	486	608	714	380	252	1440	595	703	425	273	317	333	511	370
**Met**	33.7	36.7	38.4	1110	60.5	56.7	70.2	857	760	35.8	35.1	577	39.8	62.2	62.8	24.5	38	19.9	88.9	35.3
**Orn**	176	111	62.8	121	98.8	97.6	202	177	231	92.1	141	486	141	149	237	103	74	112	164	181
**Phe**	145	111	196	130	178	184	218	240	122	174	176	392	128	158	193	166	145	114	303	197
**Pro**	219	308	174	346	47.9	246	247	403	321	201	181	1310	336	268	316	139	325	235	371	147
**Ser**	81.7	110	90.1	96.6	48.2	112	172	233	110	104	110	408	147	164	155	81.1	97.9	54.4	112	109
**Thr**	84.4	131	137	212	167	135	152	230	213	201	113	739	228	343	118	139	125	105	195	140
**Trp**	51.7	52.9	54.7	59.6	78.4	63.4	130	1	120	86.1	67.1	139	84	70.5	94.9	29.3	50.5	53.7	101	82.1
**Tyr**	83.9	78.5	127	111	55.8	88	187	170	294	117	65.6	541	115	114	92.6	72.6	157	84.8	289	121
**Val**	376	297	210	392	423	249	311	328	567	353	263	638	481	336	383	285	260	273	689	297
**Ac Orn**	1.690	0.392	0.082	0.194	0.075	0.157	0.780	0.273	0.042	0.174	0.278	1.380	0.359	0.241	0.425	0.285	0.476	0.712	0.829	0.161
**ADMA**	0.710	0.722	0.863	1.020	0.520	0.858	0.954	0.622	1.420	0.661	1.090	3.020	0.984	1.000	0.758	0.581	1.630	1.050	1.680	0.762
**Alpha AAA**	1.460	2.300	1.410	5.400	2.090	1.230	2.380	1.810	2.830	1.260	1.130	6.490	3.160	2.600	1.170	1.740	0.150	2.300	0.989	2.840
**Creatinine**	178	43.7	295	156	48.7	64.8	74.9	72.7	56.3	143	137	185	517	72.3	109	145	583	175	188	120
**DOPA**	0.155	0.205	0.174	0.212	0.140	0.236	0.438	0.434	0.399	0.203	0.176	0.583	0.201	0.230	0.231	0.132	0.040	0.092	0.356	0.223
**Kynurenine**	6.580	2.280	12.000	33.400	9.280	7.140	5.580	7.680	3.820	9.470	6.660	20.600	8.210	12.300	8.910	11.600	45.000	8.160	16.800	4.760
**Met:SO**	0.972	2.420	5.530	17.500	2.580	2.040	2.950	50.200	20.900	2.460	2.410	15.100	5.300	8.200	3.570	1.970	3.430	1.690	5.680	0.833
**Putrescine**	0.270	0.273	2.350	0.180	0.271	0.459	0.528	0.245	0.345	0.278	0.290	1.440	1.010	0.345	0.272	0.331	0.284	0.233	0.701	0.245
**SDMA**	1.220	0.150	3.650	1.150	0.150	1.150	0.774	0.901	1.320	1.440	1.780	2.540	3.300	1.710	1.680	1.370	3.340	3.870	2.190	1.110
**Spermidine**	0.124	0.311	0.156	0.093	0.058	0.060	0.437	0.132	0.060	0.249	0.108	0.199	0.161	0.065	0.044	0.049	0.089	0.041	0.103	0.201
**Spermine**	0.041	0.454	0.051	0.079	0.024	0.034	0.218	0.030	0.010	0.108	0.034	0.048	0.028	0.041	0.019	0.020	0.077	0.029	0.061	0.104
**T4 OH Pro**	8.040	6.550	9.250	36.000	10.200	6.690	9.760	11.600	18.800	13.400	5.650	161.00	6.850	12.700	7.360	5.230	11.800	5.190	24.600	4.360
**Taurine**	61.600	47.500	48.100	19.800	27.700	34.400	136.00	12.100	21.900	40.500	11.800	24.100	19.100	12.400	35.100	11.900	31.200	9.370	41.200	59.200
**Total DMA**	2.050	0.585	3.680	1.870	0.731	1.530	1.300	1.220	2.050	2.050	2.270	5.120	4.200	2.720	1.960	1.820	4.300	4.420	3.790	1.780
**SM OH C14:1**	3.052	3.027	1.895	3.372	1.232	2.229	2.219	1.520	1.262	2.860	2.290	3.114	3.159	2.455	2.112	2.186	2.986	2.252	1.257	2.093
**SM OH C16:1**	1.242	0.983	0.702	1.066	0.566	0.825	0.822	0.508	0.400	0.906	0.862	0.899	1.346	0.911	0.970	0.724	0.803	0.699	0.386	0.753
**SM OH C22:1**	0.614	0.411	0.431	0.659	0.286	0.507	0.546	0.351	0.265	0.539	0.494	0.339	0.950	0.586	0.436	0.436	0.350	0.499	0.194	0.545

**SM OH C22:2**	0.811	0.612	0.521	0.551	0.350	0.728	0.545	0.281	0.236	0.555	0.693	0.581	0.959	0.687	0.592	0.412	0.513	0.615	0.229	0.437
**SM OH C24:1**	0.076	0.018	0.057	0.061	0.008	0.033	0.086	0.051	0.025	0.037	0.040	0.080	0.070	0.061	0.032	0.041	0.044	0.045	0.023	0.035
**SM C16:0**	49.881	46.158	36.919	65.917	29.959	42.706	54.322	45.173	32.254	45.278	53.135	65.025	60.646	43.708	44.344	43.623	84.369	45.828	23.762	40.328
**SM C16:1**	6.007	6.312	4.967	7.828	3.863	6.832	6.698	4.912	4.799	8.140	7.427	9.566	10.533	6.283	6.248	5.930	10.400	6.637	2.341	4.956
**SM C18:0**	6.278	4.901	3.097	5.146	3.586	4.042	4.386	2.351	1.825	6.121	5.658	2.328	8.209	4.558	6.372	3.567	3.666	3.442	1.413	3.497
**SM C18:1**	2.688	2.184	1.322	2.107	1.622	2.112	1.856	1.076	1.136	3.219	2.768	1.420	4.639	2.178	3.151	1.774	1.842	1.947	0.591	1.583
**SM C20:2**	0.109	0.092	0.060	0.121	0.068	0.081	0.096	0.037	0.034	0.136	0.079	0.084	0.189	0.092	0.084	0.081	0.081	0.087	0.041	0.038
**SM C22:3**	0.005	0.045	0.044	0.150	0.014	0.005	0.044	0.006	0.033	0.005	0.036	0.054	0.005	0.005	0.005	0.015	0.039	0.032	0.005	0.005
**SM C24:0**	0.728	0.820	0.692	1.155	0.489	0.705	0.892	0.811	0.605	0.860	0.956	1.028	1.364	0.809	0.776	0.594	0.841	0.777	0.525	1.160
**SM C24:1**	3.269	3.244	3.025	3.294	2.030	2.800	3.004	2.477	2.491	3.166	4.045	3.913	3.571	2.449	2.476	2.409	3.726	3.823	1.756	2.590
**SM C26:1**	0.013	0.021	0.012	0.011	0.005	0.025	0.004	0.018	0.016	0.021	0.023	0.007	0.020	0.036	0.018	0.014	0.001	0.014	0.001	0.017
**C0**	71.405	32.324	58.249	270.49	17.512	25.005	115.62	55.907	110.21	17.337	52.166	112.81	75.522	49.983	59.642	69.854	101.77	66.351	110.63	28.718
**C2**	11.424	2.782	5.094	34.921	1.529	2.523	9.413	3.741	9.223	2.836	6.984	22.734	6.993	3.711	5.619	7.352	21.904	8.323	15.557	2.299
**C4**	1.058	0.645	0.622	7.272	0.227	0.632	0.767	0.263	0.441	0.272	0.524	1.371	0.765	0.520	0.699	0.374	1.543	0.949	1.686	0.784

Table S3 –Comparisons of metabolite concentrations measured at day 1 and day 7 in survivors (S) and non survivors (NS) in relationship to outcome at day 28 and 90. The values are reported as µM median (25 ,75 percentile).

	Metabolite	D1	D7	pValue	FDR
**28 day Survivors**	LPC a C16:1	0.657 (0.334, 0.970)	2.369 (1.598, 2.882)	0.042	$<10^{-6}$
	LPC a C18:2	2.005 (1.488, 3.144)	8.158 (5.488, 14.161)	0.024	$<10^{-6}$
	LPC a C20:3	0.302 (0.265, 0.456)	1.008 (0.551, 1.402)	0.042	$<10^{-6}$
	LPC a C24:0	0.054 (0.049, 0.069)	0.096 (0.086, 0.108)	0.002	$<10^{-6}$
	LPC a C28:1	0.095 (0.067, 0.115)	0.138 (0.113, 0.142)	0.014	0.029
	PC aa C30:0	2.643 (2.100, 3.107)	3.987 (3.133, 4.243)	0.010	0.002
	PC aa C32:2	19.743 (13.202, 29.768)	30.674 (22.532, 2.543)	0.032	0.004
	PC aa C32:3	2.228 (1.799, 2.920)	3.486 (2.769, 4.240)	0.007	$<10^{-6}$
	PC aa C34:1	1584.852(1220.297,2106.714)	2088.467(1951.806,2322.050)	0.010	0.005
	PC aa C34:3	51.173 (47.713, 83.221)	115.731 (85.118, 143.073)	0.001	$<10^{-6}$
	PC aa C34:4	3.737 (2.793, 5.619)	8.604 (6.879, 11.438)	0.001	$<10^{-6}$
	PC aa C36:1	119.836 (88.519, 152.720)	224.959 (153.382, 259.225)	0.001	$<10^{-6}$
	PC aa C36:3	370.840 (212.091, 423.921)	496.138 (321.304, 550.808)	0.010	$<10^{-6}$
	PC aa C36:5	47.169 (25.900, 60.058)	66.158 (49.468, 100.254)	0.003	0.004
	PC aa C36:6	1.938 (1.424, 2.404)	3.006 (2.317, 3.657)	0.001	$<10^{-6}$
	PC aa C38:0	3.923 (2.245, 4.807)	4.631 (4.197, 6.211)	0.005	0.001
	PC aa C38:1	0.757 (0.499, 0.936)	1.762 (0.880, 2.387)	0.032	$<10^{-6}$
	PC aa C38:3	70.838 (55.566, 80.983)	104.071 (71.839, 114.423)	0.024	$<10^{-6}$
	PC aa C38:6	164.022 (131.032, 174.123)	125.796 (69.163, 152.805)	0.007	0.007
	PC aa C40:2	0.587 (0.459, 0.617)	0.749 (0.554, 0.837)	0.024	$<10^{-6}$
	PC aa C40:3	0.667 (0.442, 0.746)	0.839 (0.625, 1.142)	0.032	0.002
	PC aa C40:6	35.123 (29.514, 45.323)	30.574 (14.082, 37.011)	0.014	0.011
	PC aa C42:0	0.353 (0.269, 0.474)	0.447 (0.433, 0.483)	0.032	0.001
	PC aa C42:2	0.186 (0.111, 0.221)	0.222 (0.197, 0.284)	0.019	0.009
	PC ae C30:0	0.191 (0.168, 0.249)	0.293 (0.259, 0.355)	0.001	$<10^{-6}$
	PC ae C30:2	0.050 (0.038, 0.063)	0.064 (0.055, 0.073)	0.042	0.001
	PC ae C32:1	17.536 (13.172, 20.064)	31.071 (27.207, 35.019)	0.001	$<10^{-6}$
	PC ae C32:2	4.447 (3.488, 4.934)	7.687 (6.197, 7.876)	0.001	$<10^{-6}$
	PC ae C34:0	7.193 (6.203, 8.575)	8.494 (6.944, 10.921)	0.003	0.007
	PC ae C34:1	59.345 (49.166, 83.804)	94.683 (91.369, 99.636)	0.001	$<10^{-6}$
	PC ae C34:2	30.843 (26.433, 51.014)	56.235 (46.754, 70.845)	0.002	$<10^{-6}$
	PC ae C34:3	13.078 (11.832, 21.026)	26.979 (20.127, 31.112)	0.002	0.001
	PC ae C36:1	29.252 (27.332, 35.611)	36.215 (34.598, 44.674)	0.001	0.002
	PC ae C36:2	42.687 (26.313, 50.523)	48.006 (41.721, 67.285)	0.019	0.002
	PC ae C36:3	14.781 (13.913, 22.977)	30.411 (26.438, 34.159)	0.001	$<10^{-6}$
	PC ae C36:4	36.338 (25.118, 41.717)	56.323 (49.987, 68.038)	0.005	0.002
	PC ae C36:5	24.506 (18.209, 33.656)	40.009 (31.119, 46.427)	0.005	$<10^{-6}$
	PC ae C38:0	2.633 (2.418, 3.202)	3.935 (2.199, 4.428)	0.019	0.005
	PC ae C38:2	3.782 (3.243, 4.376)	5.093 (4.571, 5.623)	0.042	$<10^{-6}$
	PC ae C38:4	21.815 (19.163, 29.019)	32.925 (27.312, 39.966)	0.005	0.001

	PC ae C38:5	26.705 (20.230, 38.512)	46.380 (42.053, 57.879)	0.003	$<10^{-6}$
	PC ae C38:6	10.553 (7.503, 12.944)	14.374 (12.127, 16.152)	0.005	0.021
	PC ae C40:1	0.911 (0.625, 1.288)	1.633 (1.209, 1.706)	0.024	$<10^{-6}$
	PC ae C40:4	2.479 (1.800, 3.645)	3.887 (3.257, 4.322)	0.032	$<10^{-6}$
	PC ae C40:6	4.665 (4.198, 5.979)	7.228 (5.338, 8.149)	0.003	$<10^{-6}$
	PC ae C42:1	0.533 (0.500, 0.654)	0.571 (0.543, 0.703)	0.032	0.016
	PC ae C42:4	0.351 (0.285, 0.519)	0.788 (0.679, 0.892)	0.001	$<10^{-6}$
	PC ae C42:5	1.353 (1.140, 2.094)	2.108 (1.848, 2.740)	0.042	$<10^{-6}$
	PC ae C44:5	0.459 (0.365, 0.642)	0.720 (0.624, 0.945)	0.003	$<10^{-6}$
	PC ae C44:6	0.462 (0.301, 0.564)	0.785 (0.650, 0.913)	0.005	$<10^{-6}$
	Sugars	6874.293(5010.346,7644.496)	8079.666(7393.151,9977.130)	0.024	$<10^{-6}$
	Arg	73.000 (42.975, 93.950)	123.000 (96.550, 220.250)	0.019	0.002
	Ser	38.300 (36.125, 137.750)	110.000 (105.250, 161.750)	0.005	0.009
	Creatinine	230.000 (131.500, 275.500)	109.000 (72.400, 167.000)	0.024	0.06
	Kynurenine	12.300 (6.287, 18.675)	7.680 (4.965, 8.735)	0.014	0.003
	Spermidine	0.076 (0.045, 0.113)	0.132 (0.061, 0.237)	0.042	$<10^{-6}$
	SM OH C24:1	0.033 (0.023, 0.049)	0.045 (0.033, 0.068)	0.032	$<10^{-6}$
	SM C16:0	35.687 (29.140, 40.569)	45.278 (43.867, 48.950)	0.001	$<10^{-6}$
	SM C24:0	0.599 (0.536, 0.710)	0.811 (0.776, 0.884)	0.002	$<10^{-6}$
	SM C24:1	1.740 (1.397, 2.737)	3.004 (2.481, 3.262)	0.014	$<10^{-6}$
	SM C26:1	0.007 (0.004, 0.012)	0.018 (0.015, 0.021)	0.019	$<10^{-6}$
	C0	32.785 (26.392, 64.080)	59.642 (36.739, 74.493)	0.024	0.039
	C4	0.325 (0.190, 0.464)	0.699 (0.461, 0.780)	0.014	0.007
**28 day Non survivors**	LPC a C16:0	10.370 (4.895, 14.712)	18.150 (14.455, 33.212)	0.083	0.001
	LPC a C16:1	0.313 (0.291, 0.591)	1.121 (0.729, 1.598)	0.042	$<10^{-6}$
	LPC a C17:0	0.134 (0.129, 0.287)	0.339 (0.277, 0.499)	0.054	$<10^{-6}$
	LPC a C18:0	1.944 (1.592, 3.925)	5.684 (3.502, 7.111)	0.102	$<10^{-6}$
	LPC a C18:1	2.778 (2.336, 4.090)	10.772 (7.079, 13.577)	0.054	$<10^{-6}$
	LPC a C18:2	2.774 (1.732, 3.356)	6.823 (4.963, 11.632)	0.024	$<10^{-6}$
	LPC a C20:3	0.299 (0.272, 0.424)	0.872 (0.469, 1.070)	0.042	$<10^{-6}$
	LPC a C20:4	0.799 (0.551, 1.330)	1.240 (0.927, 2.547)	0.147	0.003
	PC aa C34:3	64.763 (37.269, 67.939)	69.660 (53.522,130.180)	0.001	0.017
	PC aa C34:4	2.938 (2.056, 4.127)	4.150 (3.464, 5.720)	0.001	$<10^{-6}$
	PC aa C36:1	140.121(125.770,199.256)	196.723(165.908,288.444)	0.001	0.018
	PC aa C36:3	258.872(197.825,317.018)	433.000(359.777,522.048)	0.01	$<10^{-6}$
	PC aa C36:5	23.315 (18.012, 32.114)	32.121 (24.778, 41.738)	0.003	0.008
	PC aa C38:0	2.960 (2.351, 4.645)	4.617 (2.616, 7.852)	0.005	0.035
	PC aa C38:3	42.068 (37.633, 64.861)	82.570 (56.981, 90.807)	0.024	$<10^{-6}$
	PC aa C40:3	0.553 (0.355, 0.736)	0.830 (0.739, 1.355)	0.032	$<10^{-6}$
	PC ae C32:1	19.215 (15.072, 24.007)	31.845 (19.133, 42.121)	0.001	0.003
	PC ae C32:2	4.071 (3.062, 4.709)	5.572 (3.838, 7.075)	0.001	0.02
	PC ae C36:3	17.890 (15.506, 22.661)	30.503 (17.954, 34.548)	0.001	0.001
	PC ae C36:4	38.104 (24.308, 47.422)	57.318 (30.265, 84.216)	0.005	0.009
	PC ae C38:1	1.831 (0.998, 2.362)	2.663 (1.536, 3.161)	0.175	0.001

	PC ae C38:5	26.187 (20.196, 42.159)	47.061 (29.391, 76.883)	0.003	0.017
	PC ae C38:6	10.131 (6.256, 13.957)	11.101 (7.714, 20.996)	0.005	0.017
	PC ae C40:2	1.397 (1.144, 1.595)	2.052 (1.883, 2.354)	0.102	$<10^{-6}$
	PC ae C40:4	2.296 (1.881, 3.069)	3.145 (3.056, 4.077)	0.032	$<10^{-6}$
	PC ae C40:6	4.566 (2.631, 5.672)	6.216 (3.154, 9.969)	0.003	0.004
	PC ae C42:3	0.495 (0.349, 0.557)	0.648 (0.449, 0.685)	0.147	0.009
	PC ae C42:4	0.367 (0.242, 0.592)	0.608 (0.487, 0.663)	0.001	$<10^{-6}$
	PC ae C44:4	0.123 (0.092, 0.154)	0.206 (0.167, 0.268)	0.240	$<10^{-6}$
	PC ae C44:5	0.419 (0.362, 0.691)	0.694 (0.411, 0.992)	0.003	0.005
	PC ae C44:6	0.403 (0.270, 0.592)	0.452 (0.387, 0.908)	0.005	0.004
	Asn	47.100 (22.092, 78.025)	66.400 (36.150,103.275)	0.147	0.116
	Cit	10.800 (6.995, 22.850)	22.900 (14.875, 28.300)	0.175	0.067
	Glu	74.900 (51.350,115.150)	97.100 (63.075,274.000)	0.175	0.004
	Thr	76.800 (54.025,143.250)	139.000(132.500,199.250)	0.054	0.001
	Trp	24.500 (12.950, 44.250)	63.400 (53.650, 84.050)	0.067	$<10^{-6}$
	Tyr	79.800 (66.400,143.250)	111.000 (70.850,190.000)	0.102	0.023
	Putrescine	0.175 (0.113, 0.346)	0.311 (0.277, 0.950)	0.175	$<10^{-6}$
	SM OH:C22:2	0.384 (0.244, 0.453)	0.521 (0.397, 0.609)	0.320	$<10^{-6}$
	SM C24:0	0.471 (0.427, 0.582)	0.705 (0.576, 0.974)	0.002	0.001
	SM C24:1	1.865 (1.561, 2.446)	3.025 (2.315, 3.773)	0.014	$<10^{-6}$
**90 day survivors**	LPC a C16:0	20.493 (6.240, 28.505)	47.046 (30.166, 56.350)	0.012	$<10^{-6}$
	LPC a C16:1	0.657 (0.314, 0.967)	2.369 (2.038, 2.945)	0.004	0.0001
	LPC a C17:0	0.389 (0.117, 0.422)	0.709 (0.492, 1.008)	0.004	$<10^{-6}$
	LPC a C18:0	4.364 (1.679, 6.895)	10.807 (7.946, 15.048)	0.020	$<10^{-6}$
	LPC a C18:1	3.481 (1.864, 4.443)	14.485 (9.271, 19.880)	0.004	$<10^{-6}$
	LPC a C18:2	2.005 (1.450, 2.996)	8.158 (5.590, 15.761)	0.004	$<10^{-6}$
	LPC a C20:3	0.292 (0.236, 0.347)	1.008 (0.681, 1.360)	0.004	$<10^{-6}$
	LPC a C20:4	1.104 (0.583, 1.706)	2.068 (1.887, 3.014)	0.039	$<10^{-6}$
	LPC a C24:0	0.054 (0.051, 0.073)	0.102 (0.091, 0.111)	0.008	$<10^{-6}$
	PC aa C30:0	2.887 (2.184, 3.229)	4.051 (3.190, 4.391)	0.027	0.0001
	PC aa C32:3	2.629 (1.924, 3.040)	3.830 (3.359, 4.484)	0.008	0.0002
	PC aa C34:1	1661.861(1332.177,2243.574)	2314.398(1781.097,2331.305)	0.039	0.0306
	PC aa C34:3	74.940 (47.836, 87.092)	127.871 (108.386, 145.765)	0.004	$<10^{-6}$
	PC aa C34:4	5.129 (2.815, 5.778)	8.734 (8.351, 12.263)	0.004	$<10^{-6}$
	PC aa C36:1	119.836 (92.375, 160.676)	230.499 (159.540, 286.958)	0.004	0.0001
	PC aa C36:3	349.441 (205.233, 434.395)	501.059 (355.874, 588.700)	0.004	0.0032
	PC aa C36:5	51.571 (28.557, 62.269)	77.650 (51.325, 107.588)	0.004	0.0299
	PC aa C36:6	2.110 (1.630, 2.435)	3.110 (2.554, 3.759)	0.004	$<10^{-6}$
	PC aa C38:0	3.923 (2.261, 4.950)	4.631 (4.092, 6.526)	0.008	0.0013
	PC aa C38:3	70.838 (49.837, 82.160)	109.532 (73.397, 118.475)	0.008	0.0006
	PC aa C38:6	165.706 (137.786, 182.901)	125.796 (88.175, 152.147)	0.004	$<10^{-6}$
	PC aa C40:2	0.587 (0.464, 0.615)	0.761 (0.563, 0.871)	0.008	0.0004
	PC aa C40:3	0.667 (0.458, 0.705)	0.874 (0.685, 1.240)	0.004	$<10^{-6}$
	PC aa C40:6	36.449 (30.209, 48.550)	34.100 (15.915, 38.200)	0.008	0.0012
	PC aa C42:0	0.439 (0.280, 0.482)	0.449 (0.443, 0.564)	0.027	0.0014
	PC ae C30:0	0.191 (0.171, 0.266)	0.301 (0.279, 0.368)	0.004	0.0001

	PC ae C32:1	19.308 (12.970, 21.214)	32.126 (29.141, 36.308)	0.004	$<10^{-6}$
	PC ae C32:2	4.658 (3.830, 5.043)	7.789 (6.580, 8.058)	0.004	$<10^{-6}$
	PC ae C34:0	7.454 (6.794, 8.686)	8.494 (7.066, 10.538)	0.012	0.0993
	PC ae C34:1	64.332 (49.957, 89.897)	94.683 (87.988, 99.308)	0.004	$<10^{-6}$
	PC ae C34:2	39.549 (26.458, 51.424)	66.512 (45.191, 73.459)	0.008	$<10^{-6}$
	PC ae C34:3	16.469 (12.735, 24.496)	27.981 (19.421, 34.634)	0.008	0.0007
	PC ae C36:1	29.252 (25.934, 36.417)	36.215 (32.284, 46.770)	0.004	0.0048
	PC ae C36:3	20.314 (13.152, 23.485)	30.411 (25.929, 35.015)	0.004	$<10^{-6}$
	PC ae C36:4	37.012 (25.418, 44.739)	56.323 (50.261, 70.762)	0.008	$<10^{-6}$
	PC ae C36:5	25.894 (19.388, 35.713)	40.898 (35.736, 46.563)	0.008	0.0001
	PC ae C38:0	2.633 (2.293, 3.450)	3.935 (2.699, 4.504)	0.004	0.0009
	PC ae C38:2	3.782 (2.991, 4.280)	5.093 (4.526, 5.410)	0.004	$<10^{-6}$
	PC ae C38:3	8.716 (7.298, 9.934)	11.620 (10.357, 12.716)	0.020	0.0001
	PC ae C38:4	24.382 (19.702, 30.294)	32.925 (27.359, 40.637)	0.008	$<10^{-6}$
	PC ae C38:5	26.705 (23.134, 37.272)	46.380 (42.331, 57.167)	0.004	$<10^{-6}$
	PC ae C38:6	11.348 (8.556, 13.390)	14.374 (12.378, 17.236)	0.008	$<10^{-6}$
	PC ae C40:1	0.911 (0.654, 1.276)	1.669 (1.345, 1.731)	0.004	$<10^{-6}$
	PC ae C40:2	1.775 (1.520, 2.060)	2.253 (2.007, 2.331)	0.020	0.0003
	PC ae C40:4	2.479 (1.942, 3.453)	3.887 (3.151, 4.649)	0.004	0.0001
	PC ae C40:5	3.743 (3.189, 5.868)	6.705 (5.842, 8.646)	0.012	$<10^{-6}$
	PC ae C40:6	4.665 (4.241, 6.474)	7.228 (5.691, 8.613)	0.004	$<10^{-6}$
	PC ae C42:1	0.533 (0.503, 0.588)	0.571 (0.544, 0.730)	0.020	0.0002
	PC ae C42:3	0.651 (0.433, 0.695)	0.834 (0.685, 0.895)	0.039	$<10^{-6}$
	PC ae C42:4	0.336 (0.277, 0.563)	0.788 (0.640, 0.967)	0.004	$<10^{-6}$
	PC ae C42:5	1.353 (1.068, 1.924)	2.108 (1.883, 3.001)	0.008	$<10^{-6}$
	PC ae C44:4	0.153 (0.117, 0.185)	0.237 (0.210, 0.261)	0.012	$<10^{-6}$
	PC ae C44:5	0.459 (0.339, 0.683)	0.720 (0.675, 0.934)	0.004	$<10^{-6}$
	PC ae C44:6	0.462 (0.296, 0.612)	0.883 (0.747, 0.929)	0.008	$<10^{-6}$
	Ser	38.300 (35.375, 123.250)	110.000 (107.750, 157.250)	0.020	$<10^{-6}$
	Kynurenine	12.700 (9.527, 20.550)	8.160 (4.525, 9.050)	0.004	0.0046
	SM C16:0	35.687 (31.117, 39.300)	45.278 (42.863, 48.199)	0.004	$<10^{-6}$
	SM C24:0	0.599 (0.546, 0.659)	0.820 (0.777, 0.959)	0.008	$<10^{-6}$
	SM C24:1	1.740 (1.480, 2.334)	3.004 (2.487, 3.326)	0.027	$<10^{-6}$
	C0	30.205 (26.021, 72.807)	59.642 (31.423, 84.194)	0.039	0.0064
	C4	0.239 (0.179, 0.523)	0.699 (0.501, 0.771)	0.020	0.0004
**90 day non survivors**	LPC a C16:1	0.467 (0.304, 0.600)	1.121 (0.663, 1.693)	0.042	0.0004
	LPC a C18:1	2.778 (2.344, 4.281)	10.772 (6.298, 15.129)	0.042	0.0015
	LPC a C18:2	2.774 (1.645, 3.724)	6.823 (5.136, 9.972)	0.042	0.0003
	LPC a C20:3	0.331 (0.280, 0.493)	0.872 (0.427, 1.234)	0.042	0.0004
	LPC a C24:0	0.057 (0.045, 0.066)	0.075 (0.063, 0.086)	0.032	0.0018
	PC aa C30:0	2.706 (1.683, 3.136)	3.080 (2.798, 3.889)	0.019	0.0003
	PC aa C34:3	51.173(38.172, 65.232)	69.660 (54.518,120.784)	0.003	0.0060
	PC aa C34:4	2.938 (2.173, 3.918)	4.150 (3.052, 6.233)	0.010	0.0071
	PC aa C36:1	139.266(118.402,180.754)	196.723(158.202,263.745)	0.005	0.0003
	PC aa C36:3	274.335(199.691,366.813)	433.000(311.672,512.101)	0.014	0.0003
	PC aa C36:5	24.106(19.734, 33.696)	32.121 (22.740, 46.197)	0.019	0.0002

	PC aa C36:6	1.356 (0.956, 1.712)	1.796 (1.323, 2.327)	0.019	0.0023
	PC aa C38:0	2.960 (2.289, 4.371)	4.617 (3.015, 6.795)	0.014	0.0021
	PC aa C38:3	60.488(39.854, 72.910)	82.570 (52.842, 89.717)	0.042	0.0021
	PC aa C40:3	0.553 (0.359, 0.807)	0.809 (0.703, 1.226)	0.042	0.0021
	PC ae C32:1	16.370(13.753, 22.372)	27.758 (19.987, 39.248)	0.002	0.0011
	PC ae C32:2	3.726 (3.195, 4.541)	5.572 (4.072, 6.091)	0.007	0.0073
	PC ae C34:1	72.656(55.115, 92.360)	102.178 (68.687,140.645)	0.024	0.0070
	PC ae C34:2	33.998(30.948, 40.357)	52.748 (39.338, 62.845)	0.019	0.0048
	PC ae C34:3	13.514(11.361, 16.914)	18.108 (15.988, 20.480)	0.032	0.0011
	PC ae C36:3	17.322(14.259, 21.675)	30.503 (20.596, 34.276)	0.002	0.0002
	PC ae C36:4	35.296(24.735, 44.830)	57.318 (31.729, 74.544)	0.024	0.0149
	PC ae C38:1	1.831 (0.991, 2.504)	2.663 (1.503, 3.175)	0.014	0.0384
	PC ae C38:4	21.008(15.112, 23.583)	29.200 (17.373, 37.837)	0.042	0.0021
	PC ae C38:5	26.187(19.205, 42.096)	47.061 (30.967, 67.401)	0.010	0.0122
	PC ae C38:6	10.131 (6.325, 13.062)	11.101 (8.331, 19.357)	0.019	0.0270
	PC ae C40:2	1.462 (1.210, 1.705)	2.052 (1.840, 2.265)	0.032	0.0004
	PC ae C40:6	4.566 (2.800, 5.381)	6.216 (3.437, 9.323)	0.019	0.0025
	PC ae C42:4	0.379 (0.256, 0.567)	0.650 (0.523, 0.747)	0.007	0.0059
	PC ae C44:5	0.422 (0.382, 0.660)	0.694 (0.417, 0.935)	0.019	0.0015
	PC ae C44:6	0.403 (0.275, 0.552)	0.452 (0.395, 0.762)	0.024	0.0071
	Asn	47.100(31.325, 77.000)	66.400 (38.975, 90.550)	0.010	0.0116
	Cit	12.300 (7.825, 26.125)	24.000 (16.525, 33.350)	0.005	0.0070
	Glu	74.900(38.775,106.550)	97.100 (59.025,298.000)	0.007	0.0070
	Gly	183.000(155.250,248.750)	303.000(241.000,374.750)	0.042	0.0031
	Thr	76.800(53.075,165.000)	139.000(127.500,207.750)	0.003	0.0048
	Trp	24.500(14.400, 51.950)	63.400 (52.450, 94.600)	0.005	0.0011
	Tyr	79.800(55.825,153.750)	111.000 (75.425,166.750)	0.019	0.0068
	Putrescine	0.152 (0.034, 0.288)	0.290 (0.270, 0.640)	0.003	0.0004
	Spermidine	0.072 (0.036, 0.099)	0.103 (0.067, 0.130)	0.027	0.0010
	SM OH:C22:2	0.384 (0.264, 0.445)	0.521 (0.365, 0.665)	0.024	0.0022
	SM C24:0	0.471 (0.406, 0.602)	0.728 (0.618, 0.927)	0.007	0.0003
	SM C24:1	1.865 (1.529, 2.470)	3.025 (2.426, 3.618)	0.019	0.0001

Table S4 – Comparison of the absolute changes in metabolite concentrations (µM) from day 1 to day 7 (Δ=D7-D1) between survivors (S) and non survivors (NS) in relationship to outcome at 28 and 90 day. The signs mean a clear increase (↑), a clear decrease (↓), or no significant change (=).

	METABOLITE	NS		S		Pval
**28 day mortality**	LPC a C24:0	0.021 (-0.002, 0.028)	=	0.042 (0.018, 0.049)	↑	0,033
	LPC a C28:1	-0.005 (-0.025, 0.009)	=	0.036 (0.005, 0.043)	↑	0,048
	PC aa C32:3	0.212 (0.026, 0.518)	↑	1.021 (0.438, 1.556)	↑	0,028
	PC aa C38:1	-0.497 (-0.641,-0.131)	↓	1.113 (-0.001, 1.646)	=	0,010
	PC ae C32:2	1.022 (0.191, 1.867)	↑	2.657 (1.718, 2.970)	↑	0,033
	PC ae C34:3	3.259 (-0.320, 8.378)	=	10.079 (7.976,11.357)	↑	0,040
	Kynurenine	2.400 (-1.943,11.350)	=	-3.390 (-8.805,-0.265)	↓	0,019
**90 day mortality**	LPC a C24:0	0.021 (-0.001, 0.029)	=	0.042 (0.022, 0.050)	↑	0,040
	PC aa C32:3	0.212 (-0.094, 0.657)	=	1.061 (0.718, 1.902)	↑	0,012
	PC aa C34:3	18.434 (10.737, 29.888)	↑	60.933 (29.469, 77.686)	↑	0,019
	PC aa C34:4	2.092 (0.397, 2.575)	↑	4.689 (2.657, 6.184)	↑	0,048
	PC aa C38:1	-0.446 (-0.618, 0.384)	=	1.113 (-0.108, 1.6711)	=	0,040
	PC ae C32:2	1.168 (0.387, 2.368)	↑	2.679 (1.785, 3.295)	↑	0,023
	Kynurenine	1.880 (-1.500, 6.300)	=	-4.540 (-10.870, -2.245)	↓	0,003
